# Practical Guide and Review of Fossil Tip-Dating in Phylogenetics

**DOI:** 10.1093/sysbio/syaf050

**Published:** 2025-09-24

**Authors:** Nicola S Heckeberg, Alessio Capobianco, Basanta Khakurel, Gustavo Darlim, Sebastian Höhna

**Affiliations:** Department of Earth and Environmental Sciences, Paleontology & Geobiology, Ludwig-Maximilians-Universität München, Richard-Wagner-Str. 10, 80333 Munich, Germany; GeoBio-Center, Ludwig-Maximilians-Universität München, Richard-Wagner-Str. 10, 80333 Munich, Germany; Department of Earth and Environmental Sciences, Paleontology & Geobiology, Ludwig-Maximilians-Universität München, Richard-Wagner-Str. 10, 80333 Munich, Germany; GeoBio-Center, Ludwig-Maximilians-Universität München, Richard-Wagner-Str. 10, 80333 Munich, Germany; Department of Earth and Environmental Sciences, Paleontology & Geobiology, Ludwig-Maximilians-Universität München, Richard-Wagner-Str. 10, 80333 Munich, Germany; GeoBio-Center, Ludwig-Maximilians-Universität München, Richard-Wagner-Str. 10, 80333 Munich, Germany; Department of Earth and Environmental Sciences, Paleontology & Geobiology, Ludwig-Maximilians-Universität München, Richard-Wagner-Str. 10, 80333 Munich, Germany; GeoBio-Center, Ludwig-Maximilians-Universität München, Richard-Wagner-Str. 10, 80333 Munich, Germany; Department of Earth and Environmental Sciences, Paleontology & Geobiology, Ludwig-Maximilians-Universität München, Richard-Wagner-Str. 10, 80333 Munich, Germany; GeoBio-Center, Ludwig-Maximilians-Universität München, Richard-Wagner-Str. 10, 80333 Munich, Germany

**Keywords:** Divergence times, fossilized birth–death process, fossils, molecular evolution, morphological evolution, phylogeny, tip-dating

## Abstract

Phylogenetic tip-dating has been and still is revolutionizing evolutionary biology in several ways. Fossil tip-dating, where fossils are placed into a phylogeny as tips based on morphological and/or molecular character information, provides a more principled approach to infer time-calibrated phylogenies compared with node-dating. Additionally, phylogenetic trees with fossils as tips become more and more important to elucidate evolutionary processes in macroevolutionary studies (e.g., deciphering diversification patterns and directional phenotypic evolution). Fossil tip-dating is slowly gathering popularity in empirical applications and has progressed substantially since its first demonstration in 2011, with respect to improved statistical models, software, and data sets. Nevertheless, executing a phylogenetic fossil tip-dating analysis is complicated and comes with many challenges. Here, we provide an extensive review and overview of methods and models for phylogenetic tip-dating analyses with fossils. We focus both on data collection and preparation and on modeling choices. We start with a survey of all published phylogenetic tip-dating studies to date, showing common data and modeling choices as well as trends toward new approaches. Then, we walk readers through sections of molecular evolution, morphological evolution (both for discrete and continuous data), and lineage evolution (the fossilized birth–death process). In each section, we describe the data and standard models with their underlying assumptions, and provide an outlook and practical recommendations.

Reconstructing time-calibrated phylogenetic trees is essential for modern evolutionary biology, as it provides a necessary component to investigate a wide set of questions, ranging from trait evolution to drivers of diversification, from gene expression to biogeography (Matzke 2014; Smith et al. 2020; Revell and Harmon 2022; Shao et al. 2023). Inclusion of extinct species as tips in dated phylogenetic trees can drastically improve our ability to properly answer these questions (e.g., Slater et al. 2012; Betancur-R et al. 2015; Puttick 2016; Mitchell et al. 2019; Silvestro et al. 2019; Mongiardino Koch and Parry 2020; Wisniewski et al. 2022).

Over the last decade, Bayesian phylogenetic fossil tip-dating—an approach in which both the phylogenetic relationships and divergence times of extinct and extant species can be estimated simultaneously, henceforth concisely called tip-dating—has become a popular method to reconstruct dated trees (Ronquist et al. 2012; Zhang et al. 2016; Gavryushkina et al. 2017; Warnock and Wright 2020; Wright et al. 2022; Mulvey, Nikolic, et al. 2025). In contrast to phylogenetic node-dating approaches, in which only information about the oldest fossil of an extant clade is directly used to time-calibrate the tree (Ho and Duchêne 2014; but see Claramunt 2022), tip-dating can potentially include every extinct species known from the fossil record of a group of organisms, and does not require an *a priori* evaluation of the relationships between extinct and extant taxa (Ronquist et al. 2012). Furthermore, the integration of fossils as tips in a phylogenetic tree naturally allows for the inclusion of paleontological data in downstream analyses of macroevolution, such as phylogenetic comparative methods and estimation of diversification rates (e.g., Silvestro et al. 2019; Magee and Höhna 2021; Brownstein et al. 2023; Sanisidro et al. 2023; Coiro and Seyfullah 2024; Barido-Sottani and Morlon 2025). These features make tip-dating a more compelling approach than node-dating for phylogenetic and divergence time estimation.

Tip-dating is often applied to total-evidence data sets, which combine molecular data for extant (and recently extinct) species with morphological data for extant and extinct species (Ronquist et al. 2012; Zhang et al. 2016; Gavryushkina et al. 2017; Mulvey, Nikolic, et al. 2025). However, tip-dating approaches can also be applied to morphological data sets alone, sampling either living and fossil organisms (Field et al. 2020) or only fossils in the case of organisms that are completely extinct (Sanisidro et al. 2023; Pol et al. 2024); to molecular data sets alone, with extinct species either represented by ancient molecules (Welker et al. 2020; Paterson et al. 2024) or devoid of character data but with phylogenetic constraints (Heath et al. 2014); or even to a combination of other data types such as geographic and ecological data (Landis et al. 2021). This flexibility derives from the modular nature of Bayesian tip-dating analyses, which integrate different data sources through shared model parameters, such as tree topology and age of origin of the tree. In general, a tip-dating analysis consists of several components that can be modeled independently (Fig. 1). The main components of a tip-dating analysis typically include the character models (molecular substitution model and morphological evolution model), the clock models (molecular and morphological), and the lineage evolution model—often called the tree model (Warnock and Wright 2020; Mulvey, Nikolic, et al. 2025).

**Figure 1.**
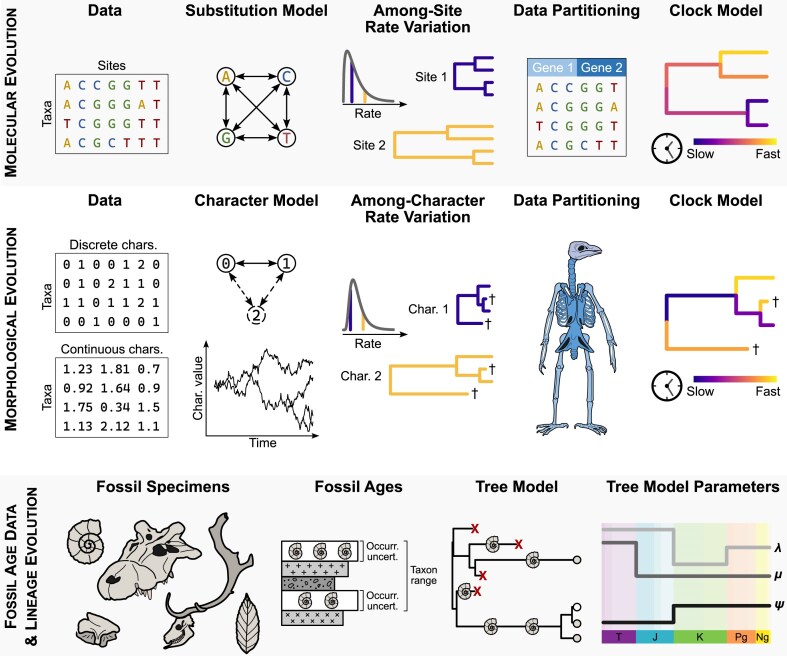
Overview of the main components involved in a Bayesian phylogenetic tip-dating analysis. This overview presents the key concepts, which we elaborate on in detail in the main text. Rows correspond to the three main sections of this paper: molecular evolution; morphological evolution; and fossil age data and lineage evolution. Panels within each row refer to aspects of a Bayesian fossil tip-dating analysis that are discussed within each section. Dagger symbol (†) in phylogenies indicates extinct taxa. “Bird man” silhouette illustrating morphological data partitioning adapted from Belon du Mans (1555).

Despite the obvious appeal of tip-dating approaches and the rapid growth of studies applying them in the last 10 years, conducting a Bayesian tip-dating analysis remains a very challenging task for both practical and theoretical reasons. The complexity and hierarchical structure of these models create strong parameter interactions and dependencies, which makes tip-dating analyses computationally intensive. This might translate into practical limits of data set size (both in terms of number of species and number of characters) that can be analyzed under this approach. In contrast, some components of a tip-dating analysis—including morphological data and the amount of sampled extinct species—might not carry enough information to reliably estimate key parameters, such as topology with fossils, or extinction and fossilization rates (Barido-Sottani et al. 2020; Warnock et al. 2020). Additionally, despite considerable scientific effort toward this direction, models employed in tip-dating analyses might still be extremely unrealistic, or make assumptions that are strongly violated by empirical data sets (Wright et al. 2016; Goloboff et al. 2019; Matschiner 2019; Beaulieu and O’Meara 2023).

Here, we provide a state-of-the-art overview of Bayesian tip-dating by breaking down a tip-dating analysis into all its components, summarizing core concepts and recent modeling advances, and outlining general recommendations and practical guidelines for each of them. Furthermore, we survey all empirical tip-dating studies to date to identify common practices and potential mismatches between theory and empirical applications. Finally, we highlight open questions that we believe should be prioritized by the community for further advancement of the field.

## General Overview

The main components of a tip-dating analysis, sorted by data types, are highlighted in Figure 1. In the following sections, we first look at molecular data, focusing on: Multiple Sequence Alignments (MSAs); models for molecular sequence evolution (including substitution models, Among-Character Rate Variation [ACRV], and data partitioning); and molecular clock models. Then, we address morphological data, delving deep into: models for morphological character evolution for both discrete and continuous characters; partitioning approaches; and morphological clock models. The last main section is dedicated to temporal information provided by geological and paleontological data, to how that information is included in a tip-dating analysis; and to lineage evolution (tree priors) modeling diversification and sampling through time, with a focus on the fossilized birth–death (FBD) model and its extensions.

### Literature Survey of Empirical Tip-Dating Studies

We conducted a literature survey in order to gather information about tip-dating analyses on empirical data sets (see also Mulvey, Nikolic, et al. 2025). We used Google Scholar to look at all publications citing a selection of foundational tip-dating studies (Pyron 2011; Ronquist et al. 2012; Gavryushkina et al. 2014, 2017; Heath et al. 2014; Zhang et al. 2016; Stadler et al. 2018), and among those, we identified publications performing tip-dating analyses on empirical data sets. We compiled relevant information—including software of choice, data set size, types of data used, and clock and tree models employed—in a table (Supplementary Table S1, available on Dryad: https://doi.org/10.5061/dryad.gb5mkkx03). When it was possible, this information was extracted directly from data and script files provided as supplementary material of each publication; otherwise, we used the information written in the methodological sections of main and supplementary texts.

While we originally included tip-dating studies without morphological data in which either the whole topology was fixed (e.g., Wisniewski et al. 2022) or topological constraints were applied to extinct taxa (e.g., Heath et al. 2014), we pruned those studies from our survey before downstream analyses for summary figures and statistics. This is because we wanted to focus on studies that use tip-dating as an analytical approach to co-estimate the topological position of extinct taxa with divergence times, rather than just estimating divergence times on an *a priori* fixed topology, or pruning extinct tips afterwards to obtain a dated phylogenetic tree of only extant species.

Figure 2, Supplementary Figures S1–S9, and Supplementary Table S1 summarize relevant components of tip-dating analyses to date. The results of the literature survey will be used to discuss common practices and potential pitfalls of empirical tip-dating analyses throughout the sections of this paper.

**Figure 2.**
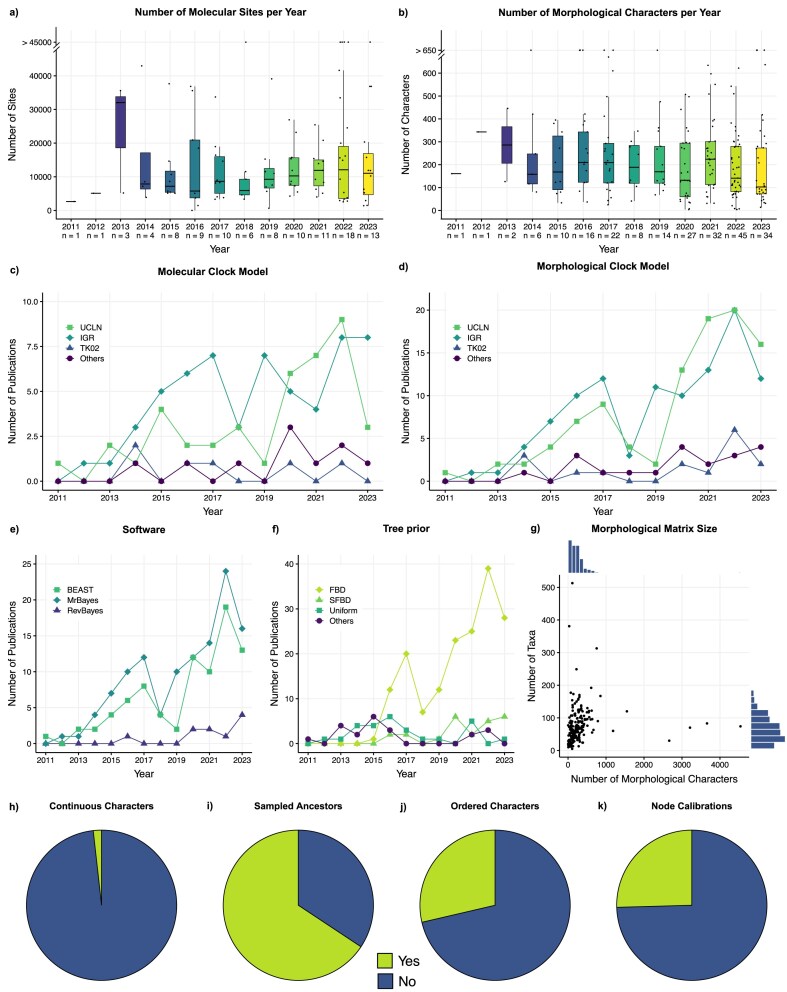
Overview and summary of tip-dating studies. These plots are based on Supplementary Table S1 containing the statistics of tip-dating studies from 2011 to 2023. (a) and (b) Box plot illustrating the number of molecular sites and morphological characters used in tip-dating studies for each year. The *x*-axis also denotes the total number of studies for that specific year, with each study represented by a dot. “> 45000” and “> 650” indicate the studies that contain more than 45,000 sites and more than 650 characters, respectively. (c) and (d) Plots showing the use of different types of molecular and morphological clocks per year. (e) Software choice for tip-dating studies per year. Software that has been used less than five times in total has been excluded from this plot for clarity. (f) Choice of tree prior per year. (g) Marginal plot showing the number of taxa and the number of character in morphological matrices used in tip-dating studies. (h–k) Pie charts showing the use of continuous characters, the presence of sampled ancestors in the analyses, the use of ordered characters, and the use of additional node calibrations in tip-dating studies, respectively. Additional plots summarizing the literature survey can be found in the Supplementary Material (available on Dryad: http://dx.doi.org/10.5061/dryad.gb5mkkx03).

## Molecular Evolution

Molecular sequence data remain the most prevalent source of information for phylogenetic analyses—including tip-dating—as genetic data provide the most easily accessible and information-rich data source for living organisms (Yang and Rannala 2012; Nascimento et al. 2017; Warnock and Wright 2020; Wright et al. 2022; Mulvey, May, et al. 2025). Modeling molecular sequence evolution has a long history in phylogenetics and is nowadays well established with several mature models and methods (see Yang and Rannala 2012 for a review). Importantly, despite the recent transition to phylogenomic studies, that is, phylogenetic studies that use genomic data containing hundreds of thousands of nucleotides from up to thousands of loci (Kapli et al. 2020; Lozano-Fernandez 2022), tip-dating studies primarily use multilocus molecular data sets with a total of few thousands to 50,000 base pairs for computational efficiency (Fig. 2a). Thus, we will focus our discussion of phylogenetic models of molecular evolution on smaller-sized multilocus data sets instead of full genomic molecular data.

### Data Selection and Considerations for Tip-Dating

The foundation of any reliable tip-dating analysis lies in the meticulous selection of molecular sequences (Philippe et al. 2011). The quality, type, and scope of these data profoundly influence not only the accuracy of the inferred phylogenetic tree topology but also the estimation of branch lengths (Lemmon and Lemmon 2013). Ill-suited or poorly curated data sets can introduce systematic biases, substantial uncertainties, leading to misleading age estimates and hampering downstream macroevolutionary inferences (Philippe et al. 2011; Dávalos et al. 2012). Therefore, careful consideration of data properties in relation to the specific goals of the tip-dating study—such as the timescale of interest, the diversity of the focal clade, and the availability of fossil calibration—is pivotal for generating credible and insightful results.

For molecular data, the choice of genetic markers is a critical decision that requires balancing several factors (Simon et al. 2006; Lemmon and Lemmon 2013). There is no universally “best” set of markers; suitability depends heavily on the evolutionary depth of the phylogeny being reconstructed and the specific questions being addressed (Faircloth et al. 2012; Townsend et al. 2012). For instance, rapidly evolving markers such as mitochondrial regions or nuclear introns might be informative for recent divergences among closely related species or populations, providing sufficient variation to resolve shallow nodes and estimate short branch lengths. Conversely, more conserved markers, such as nuclear coding regions (exons) or Ultra-Conserved Elements (UCEs), are often preferred for deeper divergences, as they are less prone to saturation, which can obscure ancient relationships and distort branch length estimates (Philippe et al. 2011; Faircloth et al. 2012; McCormack et al. 2013). Besides, for ancient divergences, the use of amino acid sequences can prove vital (Jeffroy et al. 2006; Philippe et al. 2011). The ideal data set for tip-dating will often comprise a strategic combination of markers that collectively provide strong phylogenetic signal across the entire temporal scope of the study, while minimizing known sources of systematic error (Salichos and Rokas 2013).

### Multiple Sequence Alignments

Molecular sequence alignments play a fundamental but often overlooked role in tip-dating analyses. As a first step, one needs to process the unaligned sequences into an MSA. Most importantly, an MSA is a homology statement (i.e., a hypothesis of homology) for each nucleotide site (Bromham 2019), used downstream to model nucleotide substitutions. Several standard tools are available for molecular sequence alignment, including (1) Clustal (Higgins and Sharp 1988), (2) MAFFT (Katoh et al. 2002), (3) MUSCLE (Edgar 2004, 2022), (4) T-Coffee (Notredame et al. 2000), and (5) MACSE (Ranwez et al. 2011, 2018).

Depending on the type of molecular data examined, alignment methods can be broadly classified into three classes: alignment by nucleotide, alignment by codon, and alignment by amino acid. Alignment by nucleotide compares DNA sequences at the nucleotide level, revealing point substitutions, and insertions and deletions (indels). Alignment by codon compares DNA triplets that code for specific amino acids, identifying changes in the coded amino acid and frame shifts (Bininda-Emonds 2005; Ranwez et al. 2011, 2018). Lastly, alignment by amino acid compares protein sequences based on their constituent amino acids, providing valuable insights into protein function and predicting conserved motifs (Abascal et al. 2010). Alignments by codon and amino acid can only be used for protein-coding gene sequences, and introduce indels only by blocks of three nucleotides (or one amino acid). In empirical tip-dating studies, the majority of molecular data sets consists of protein-coding DNA sequences (Supplementary Fig. S3). Thus, alignments by codon or amino acid should be preferred to alignments by nucleotide when aligning protein-coding regions, as they avoid point indels that might cause a shift in the reading frame of the sequence, for example, introducing spurious stop codons.

While foundational, the impact of alignment strategy and quality can be profound for phylogenetic analyses, potentially impacting the results as much as any other components of the analysis (e.g., taxon and character sampling, model and prior specifications) (Talavera and Castresana 2007; Zhang, Qin, et al. 2023). Misaligned sequences can lead to erroneous estimations of phylogenetic relationships and consequently impact divergence time inferences (Bromham et al. 2018). However, the specific influence of alignment methods and misalignment on tip-dating studies remains underinvestigated.

Even though sequence alignment is performed semi-automatically through algorithm-based tools, additional manual editing and visual checks are most often applied in preparing a data set for downstream applications. Several software tools exist for filtering and polishing alignments without having to rely on nonreplicable manual edits, including (1) Gblocks (Talavera and Castresana 2007), (2) trimAl (Capella-Gutiérrez et al. 2009), (3) ClipKit (Steenwyk et al. 2020), (4) CIAlign (Tumescheit et al. 2022), and (5) alignmentFilter (Zhang, Qin, et al. 2023).

#### Open questions

Although the traditional approach separates multiple sequence alignment (MSA) and phylogenetic estimation, methods to simultaneously estimate MSA and phylogeny have been explored (Fleissner et al. 2005; Lunter et al. 2005; Redelings and Suchard 2005; Suchard and Redelings 2006). The applicability and efficacy of these simultaneous alignment and phylogenetic estimation methods within the context of tip-dating warrant further investigation. It is likely that joint inference of alignment and phylogeny is computationally not feasible, and alternative approaches such as alignment averaging would be a fruitful compromise (Ashkenazy et al. 2019).

**Figure 3.**
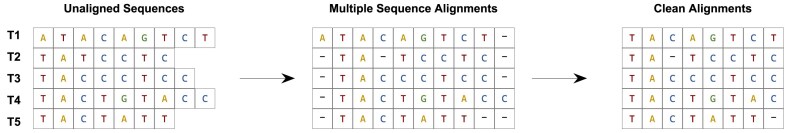
A schematic showing the process of obtaining molecular sequences. *Unaligned Sequences*, as the name suggests, are the raw reads of DNA obtained from various sequencing approaches. The raw sequences are then aligned into *Multiple Sequence Alignments* by establishing homology using various algorithms. As a result, the algorithms insert gaps between the nucleotides. After this process, we can optionally “refine” the multiple sequence alignment to remove any problematic regions and thus obtain the *Clean Alignments*.

Manual sequence alignment editing remains a prevalent practice to achieve refined sequence data (see Fig. 3). However, this approach compromises the reproducibility of scientific investigations. In addition to that, some studies have shown that manual filtering might induce potentially negative downstream effects (Tan et al. 2015; Ranwez and Chantret 2020). Consequently, the development of automated and reliable alignment filtering pipelines is crucial to enhance the replicability of research endeavors. The impact of misaligned sequences on a tip-dating analysis is currently unknown and needs to be further explored. In that respect, it might be interesting to apply alignment error models for phylogenetic inference as terminal branches are particularly impacted by sequencing errors (Chen et al. 2022; Kozlov et al. 2022).

#### General recommendations

To ensure the accuracy and reproducibility of phylogenetic studies, meticulous curation of MSAs is crucial. We suggest the following practices to perform MSA:

1. Alignment software: If aligning noncoding regions, use of the software T-Coffee or MAFFT recommended based on accuracy (Pais et al. 2014), or use of codon or amino acid alignment methods when aligning protein-coding sequences (MACSE, Ranwez et al. 2018).
2. Automatic filtering tools: Software such as alignmentFilter (Zhang, Qin, et al. 2023) and CIAlign (Tumescheit et al. 2022) streamlines the MSA filtering process, enhancing the reproducibility of the study.
3. Data accessibility: Both the final curated MSA as well as the original unaligned sequences is uploaded as research-associated data.

### Models for Molecular Sequence Evolution

Molecular evolution is commonly modeled using substitution models, which belong to continuous time Markov chains (CTMC). A fundamental assumption of a CTMC is the Markov property: if a Markov chain is in state *i* at time $t_0$, then the probability of change from *i* to state *j* after time $\Delta t$ depends only on the current state *i* (in other words, the process is memory-less). Furthermore, substitution models assume that each site in the sequence evolves independently and is identically distributed. Because most empirical tip-dating studies use DNA rather than amino acid data (Supplementary Fig. S3), we focus the discussion about molecular sequence evolution primarily on DNA. However, similar principles as discussed here can be applied to models of amino acid evolution (Yang et al. 1998; Huelsenbeck, Joyce, et al. 2008).

In general, the substitution model assumes an exponential waiting time for a substitution (or transition) from state *i* to state *j*, which is governed by the substitution rate $r_{ij}$. Thus, a substitution model is described by the instantaneous rate matrix *Q*


(1) \begin{eqnarray*}
\mathcal {Q}_{ij} = {\begin{pmatrix}
- &\quad r_{AC} &\quad r_{AC} &\quad r_{AT} \\
r_{CA} &\quad - &\quad r_{CG} &\quad r_{CT} \\
r_{GA} &\quad r_{GC} &\quad - &\quad r_{GT} \\
r_{TA} &\quad r_{TC} &\quad r_{TG} &\quad - \\
\end{pmatrix}},
\end{eqnarray*}

which describes the rates of change between the nucleotides. Here, we use the rate $r_{AC}$ in a general form to represent the substitution rate from an “A” to a “C,” and provide specific alternative examples in Supplementary Table S2. The *Q*-matrix is used in calculating the transition probability matrix (Moler and Van Loan 1978, 2003), which in turn is used to calculate the probability of the observed nucleotide sequences at the tips of the phylogeny (Felsenstein 1981). The diagonal elements in the matrix are specified such that the rows sum to zero. The rate matrix is rescaled such that the mean rate of substitution equals to one. This rescaling allows us to interpret the branch lengths as the expected number of substitutions per site, or to apply specific molecular clock models (see below).

There are several different time-reversible substitution models commonly used in molecular evolution that differ in how the rates of the substitution matrix *Q* are defined (Supplementary Table S2). Although we focus here on time-reversible models, there are numerous non-time-reversible models that can potentially be defined (Yang 1994; Huelsenbeck et al. 2002; Williams et al. 2015). The rate matrix depends on two key parameters. First, the *stationary frequencies* represent the relative abundance of each nucleotide (A, C, G, and T) within a sequence. Depending on the substitution model used, the stationary frequencies can either be fixed to equal values, estimated as a parameter of the model, or specified prior to the analysis based on the frequencies of the empirical data (Whelan et al. 2001). Second, the *exchangeability rates* quantify the rates of changing from one nucleotide to another. By incorporating these parameters in different combinations, substitution models provide a powerful tool for analyzing molecular sequences. The simplest substitution model is the Jukes–Cantor (JC69) model (Jukes and Cantor 1969) without any parameter (because the rate matrix is always rescaled and the rate of evolution enters through the branch length) and assumes equal frequencies and transition rates between any character state, whereas one of the most commonly used complex models is the General Time Reversible (GTR) model (Tavaré 1986; Rodriguez et al. 1990), containing eight free parameters. The GTR model allows for rates of substitution of each pair of nucleotide to be different and also assumes different stationary frequencies of nucleotides, thus much more flexibility in the assumptions about the sequence evolution. Other commonly used models are the K80 (Kimura 1980), HKY (Hasegawa et al. 1985), and F81 (Felsenstein 1981) models, all of which relax some of the assumptions of the JC69 model without reaching the complexity of the GTR model in terms of number of free parameters.

#### Among-character/site rate variation

In addition to the substitution rate matrix, there are some extensions that can be applied to the substitution process to incorporate rate heterogeneity among sites/characters (Yang 1994). The rate of nucleotide substitutions can vary from site to site in the sequence. For example, in protein-coding sequences, the third nucleotide position of a codon evolves faster than the first two positions, and thus is more variable (Shapiro et al. 2006). This can be accounted for by using a distribution of rates over sites. A common approach for this is to use a discrete gamma model, indicated by $+\Gamma$ (also $+G$ or $+\mathrm{GAMMA}$) (Yang 1994). In this approach, several categories of rates (usually four but see Susko et al. 2003; Mayrose et al. 2005; Jia et al. 2014) are used to approximate the gamma distribution with equal probability for each category. The rates per site are regarded as random variables drawn from a discrete distribution and summed over in the probability computation.

The presence of invariable sites in the sequence has also been known to impact the estimation of genetic divergences (Steel et al. 2000). To account for invariability of sites, a proportion of site is considered invariable. This is known as the *Invariable sites model* ($+I$). This extension allows for any given site to be variable or invariable (Churchill et al. 1992; Reeves 1992; Sidow et al. 1992). However, a combination of the $+\Gamma$ and $+I$ model can lead to non-identifiability of parameters (Yang 2014, p. 120).

#### Partition models

**Figure 4.**
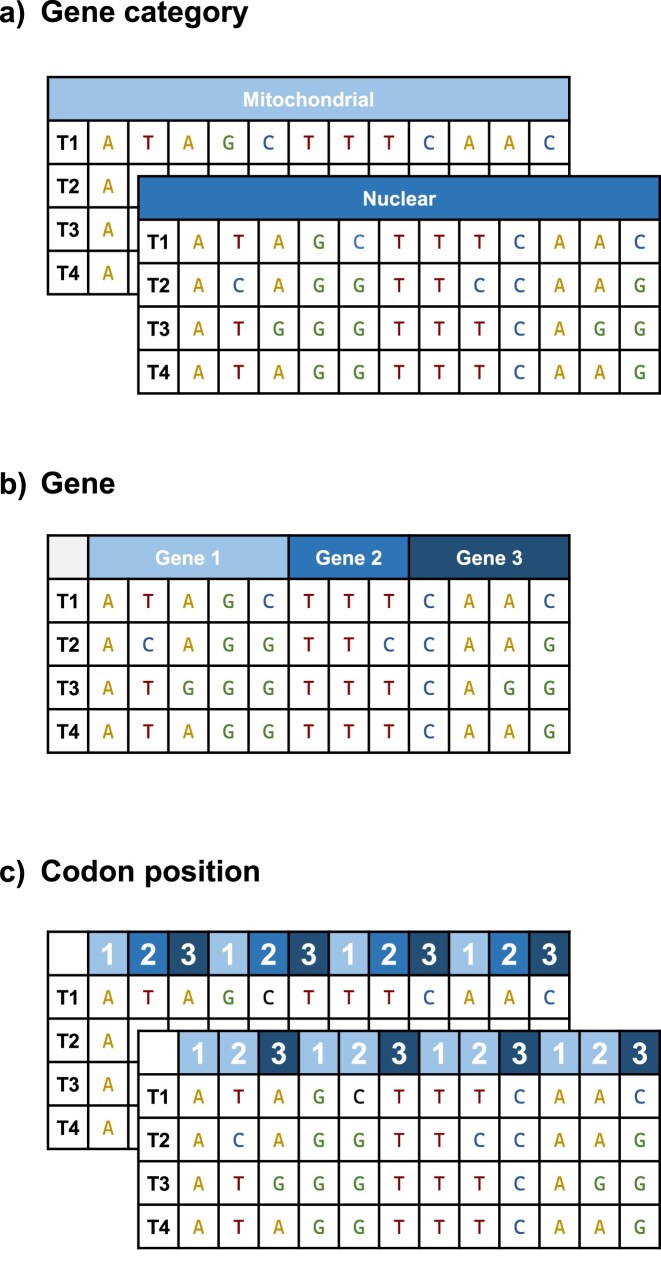
Partitioning molecular data. This figure shows different partitioning schemes for molecular data. (a) The broadest partitioning scheme is by gene category, for example, nuclear and mitochondrial markers. (b) A more detailed partitioning scheme comprises each gene or locus as a separate data subset. (c) Coding sequences are often partitioned by codon position, where either each position represents a separate data subset or codon positions 1 and 2 form one data subset and position 3 forms another.

To further account for heterogeneity in the data set, molecular data can be partitioned in several data subsets (Bull et al. 1993; Kainer and Lanfear 2015). In general, the approach on how to partition the data into subsets is variable and depends on the specific type of available data. At the highest level, the data can be divided by *gene category*, for example, into mitochondrial and nuclear data as, in general, the mitochondrial loci and nuclear loci have different evolutionary tempi (Fig. 4a). The next level of data partitioning is commonly done by *locus* or *gene*, and the most small-scaled partitioning is done within a gene or locus, for example, by *codon position* (Fig. 4c; Shapiro et al. 2006). Note that the correct terminology defines a *partition* as the specific subdivision of the whole data set into data subsets, although in the phylogenetics community a data subset is often improperly called a partition.

In theory, the evolutionary process for each data subset could be modeled with its own substitution model (e.g., GTR vs. HKY) with its own substitution model parameters (stationary frequencies and exchangeability rates). Alternatively, some parameters could be linked, that is, they are assumed to be shared and therefore identical for different data subsets. Specifically, the substitution model, the individual branch lengths (and tree topology), and the overall substitution rate can be estimated separately for each data subset. For example, some data subsets might have a higher guanine-cytosine content (GC-content) and therefore are better modeled with different stationary frequencies, whereas the exchangeability rates of the underlying substitution process could be assumed to be identical. Similarly, some data subsets evolve faster or slower and therefore often require their own rate multiplier (also known as partition rate multiplier) (Yang 1996; Marshall et al. 2006; Fan et al. 2011). The selection of rate multipliers and partitioning strategies can significantly influence the accuracy and precision of divergence time estimates. The flat Dirichlet distribution (or sometimes a more generalized symmetric Dirichlet distribution) is the prevalent choice for the distribution of partition rate multipliers (Marshall et al. 2006).

The simplest partition model is the one where all parameters are linked between all data subsets, that is, all sites in the alignment evolved under the same process as described by the shared tree, branch rates, and parameters of the substitution model. The most complex partition model is the one where each data subset has its own independent substitution model, substitution model parameters, and branch rates. In between these two extremes lie many possible models (their number grows more than exponentially with the number of data subsets).

When setting up a partition model, the user can specify the extent to which the model parameters are shared between the data subsets. Software such as PartitionFinder (Lanfear et al. 2012, 2017) helps in partitioning the data sets into several subsets and also suggests the best substitution model for each data subset. However, since the number of possible data partitioning schemes is excessively large, complicated by the fact that the best fitting partitioning scheme depends on the actual phylogeny, automatic tools such as PartitionFinder perform greedy heuristic algorithms. Alternatively, infinite mixture model approaches integrate over all possible partitioning schemes (Lartillot and Philippe 2004; Wu et al. 2013), but are often restrictively slow. Recently, it has been shown that Bayesian inference of phylogeny is robust to substitution model overparameterization and assigning a separate GTR+$\Gamma$ substitution model per data subset, albeit computationally more intensive, can be an adequate alternative (Fabreti and Höhna 2023).

#### Open questions

Complex substitution models such as non-reversible substitution models (Yang 1994; Huelsenbeck et al. 2002), non-stationary substitution models (Williams et al. 2015), covarion-like substitution models (Galtier 2001), and Markov Modulated Models (Baele et al. 2021) can increase realism of substitution model to empirical DNA data. Despite the long history of these more complex substitution models, neither has been applied in tip-dating studies. In general, the application of more complex substitution models beyond standard DNA substitution models holds promise for enhancing the robustness of tip-dating studies, although significant computational resources may be required.

Our literature survey revealed a notable scarcity in the application of amino acid data sets for tip-dating analysis (6 out of 182). Amino acid data sets can be utilized for resolving deep evolutionary divergences where nucleotide data might suffer from saturation and morphological data are scarce (Philippe et al. 2011). Several factors may have contributed to the trend of less amino acid data usage. One of the factors could be the relative ease of obtaining nucleotide data sets. The selection of appropriate loci and the bioinformatics involving the processing of those data sets can be a potentially difficult task than obtaining nucleotide data sets from public databases. Additionally, the computational demands of employing empirical amino acid substitution models (e.g., PAM [Dayhoff et al. 1978], JTT [Jones et al. 1992], LG [Le and Gascuel 2008], WAG [Whelan and Goldman 2001]) or amino acid mixture models (Lartillot and Philippe 2004;Le et al. 2008; Schrempf et al. 2020), which are inherently more complex and parameter-rich than nucleotide models, can also be a potential barrier. The neglected use of amino acid substitution models could be more appropriate for the deep timescales often considered in tip-dating studies (Lozano-Fernandez 2022).

The most common approach to model rate multipliers for different partitions uses a flat Dirichlet distribution. Other priors, such as gamma or lognormal distributions with a discrete number of categories, if appropriately applied with estimated mean rates (dos Reis et al. 2014), might greatly reduce computational time in partitioned data sets with a high number of subsets. Exploration of these other options for rate multipliers might be a promising area of research.

#### General recommendations

To ensure clarity and reproducibility, we recommend the following practices for model specification:

1. Substitution model choice: Substitution models can be chosen automatically via software such as ModelFinder (Kalyaanamoorthy et al. 2017) or PartitionFinder (Lanfear et al. 2017). Alternatively, because Bayesian inference of phylogeny is robust to model overparameterization (Fabreti and Höhna 2023), a separate GTR+$\Gamma$ substitution model can be applied to each data subset without overfitting and the requirement of model selection.
2. Partitioning approach: We suggest to partition the data set *a priori* by locus and by codon position (in case of protein-coding loci). Then, either a tool such as PartitionFinder (Lanfear et al. 2017) should be used especially to merge data subsets (e.g., when these are small with fewer than 100 sites) to ensure computational tractability, or to apply an independent GTR+$\Gamma$ substitution model to each data subset. Importantly, among partition rate variation should be incorporated into the model.
3. Increased transparency in decision-making: Comprehensive documentation of the rationale underlying all modeling decisions facilitates the reproducibility of the study by other researchers.

### Molecular Clocks

The molecular clock hypothesis has transformed the field of phylogenetics by enabling inference of time-calibrated phylogenies from molecular data (Donoghue and Yang 2016; dos Reis et al. 2016). The hypothesis states that substitutions along a lineage occur at a constant pace, exhibiting a “clock-like” behavior (Zuckerkandl and Pauling 1962, 1965). Thus, the number of substitutions on a branch is proportional to time and clock rate. The assumption of a molecular clock enables inference of divergence times by transforming branch lengths in units of substitution per site into actual time, using the following equation:


(2) \begin{eqnarray*}
\text{branch length} = \text{clock rate} \times \mathrm{time}.
\end{eqnarray*}

**Figure 5.**
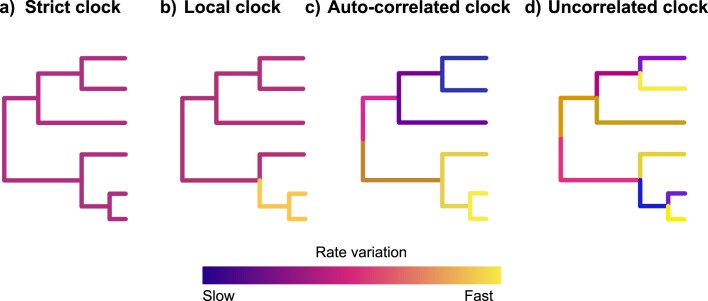
Representation of rate variations in topologies under different molecular clock models: (a) the strict clock assumes constant rate of substitution across the tree, (b) local clock models allow distinct rates in a given region of the tree, (c) the autocorrelated clock assumes that rates of evolution in descendant branches derive from the ancestor’s branch, and (d) the uncorrelated clock allows independent rates of substitution across the tree.

Substitution rates can be influenced by a variety of factors such as generation times, population sizes, and metabolic rates (Ohta and Kimura 1971; Gillespie 1991). Moreover, it has been shown that branch lengths measured in substitutions vary across the phylogeny, that is, there is a different root-to-tip distance for different extant taxa (Langley and Fitch 1974). Thus, assuming a constant rate for a molecular clock is almost never warranted, except perhaps in very closely related taxa. To accommodate this variation and to relax the molecular clock, several *relaxed clock* models have been suggested (Table 1 and Fig. 5; Thorne et al. 1998; Huelsenbeck et al. 2000; Aris-Brosou and Yang 2002; Thorne and Kishino 2002; Drummond et al. 2006). The most common relaxed clock models can be categorized into (1) autocorrelated relaxed clock (e.g., ACLN, TK02, and CPP; Huelsenbeck et al. 2000; Kishino et al. 2001; Thorne and Kishino 2002), (2) uncorrelated relaxed clock (e.g., Independent Gamma Rate [IGR], Uncorrelated Exponential Clock [UCE], and Uncorrelated Log-Normal Clock [UCLN]; Drummond et al. 2006), and (3) mixture models (e.g., random local clock, Dirichlet process prior [DPP], and mixtures of uncorrelated and autocorrelated clocks; Drummond and Suchard 2010; Heath et al. 2012; Darlim and Höhna 2024). Although few empirical tip-dating studies have tested different relaxed clock models and their fit to the molecular data set used (e.g., Lartillot et al. 2016; Simões, Vernygora, et al. 2020), most studies pick only one clock model *a priori*. Our literature survey points out that the choice of relaxed clock model seems to be highly correlated with the choice of software used for the tip-dating analysis, with the IGR model favored by MrBayes users and the UCLN model favored by BEAST users (Fig. 2; Supplementary Table S1). This suggests that most often the choice of clock model is not dictated by properties of the data set used or considerations about molecular rate heterogeneity in the focal clade, but instead by model availability and “default practices” in the software of choice.

**Table 1.** Short listing of commonly used clock models in tip-dating studies

Clock model	Description	References
Global molecular clock	Rate of substitution over time is constant across the tree (strict clock).	(Zuckerkandl and Pauling 1962, 1965)
Local molecular clock	Different regions of the tree can have different rates but closely related lineages have identical rates, for example, random local clock or Dirichlet process prior (DPP) clock.	(Yoder and Yang 2000; Drummond and Suchard 2010; Heath et al. 2012)
Compound Poisson process	Rate changes according to a point process, and new rates are the product of old rate and $\Gamma$ distributed multiplier (Punctuated Rate Change Model).	(Huelsenbeck et al. 2000)
Autocorrelated clock	Rate of evolution in a descendant branch arises from a probability distribution centered on the rate of the ancestor’s branch, for example, using Brownian motion, Ornstein–Uhlenbeck, or Cox–Ingersoll–Ross processes.	(Kishino et al. 2001; Aris-Brosou and Yang 2002; Lepage et al. 2006)
Uncorrelated clock	Rate of a particular branch is not dependent on the rates of neighboring branches or the ancestral branch. Several uncorrelated clocks are common in tip-dating studies, namely, Uncorrelated Log-Normal Clock (UCLN), Independent Gamma Rate (IGR), and Uncorrelated Exponential Clock (UCE).	(Drummond et al. 2006; Lepage et al. 2007; Rannala and Yang 2007)

#### Open questions

Exploration of the practical impact of different molecular clock models has mostly focused on divergence time estimates on a fixed topology in a node-dating framework (Duchêne, Lanfear, et al. 2014; dos Reis et al. 2018; Nie et al. 2020). It is unclear if and how different molecular clock models can impact topological estimates, and how these differ from unrooted, nonclock topologies. Early investigations in this direction have reported conflicting results (Drummond et al. 2006; Wertheim et al. 2010).

Molecular clocks in a Bayesian framework allow to infer the root of a phylogenetic tree (Huelsenbeck et al. 2002). This is particularly useful when the tree cannot be rooted *a priori* by including obvious outgroups to the group of interest (Boykin et al. 2010; Calvignac-Spencer et al. 2014). Additionally, including outgroups in a tip-dating analysis can drastically alter the sampling strategy of the study, biasing diversification rate and divergence times estimates (see the “The Fossilized Birth–Death Process” subsection). However, the accuracy of molecular clock rooting has been tested only for very limited cases—few tips and moderate variance in clock rates (Huelsenbeck et al. 2002; but see Tria et al. 2017 for poor performance of molecular clock rooting on more complex empirical cases). Simulation studies evaluating the performance of molecular clock rooting under different clock models and rate heterogeneity scenarios are needed to establish the conditions under which it is necessary (or not necessary) to specify the root of the tree *a priori*.

As relaxed clock models are parameter-heavy, adding independent clocks to model the rates of molecular evolution for different loci comes at a weighty computational cost (Duchêne, Molak, et al. 2014). This is why most empirical tip-dating studies use only one or two (e.g., mitochondrial clock + nuclear clock) independent molecular clocks (Supplementary Table S1). If the analyzed loci differ only in mean rates of molecular evolution, but not in among-lineage rate variation, then partition rate multipliers rather than multiple independent clocks should be sufficient to properly model molecular rates across several loci (see Zhu et al. 2015; Foster and Ho 2017; Duchêne et al. 2020). The R package ClockstaR identifies the optimal number of independent clocks for a data set and its best clock-partitioning strategy by looking at the patterns of among-lineage rate variation for different loci (Duchêne and Ho 2014; Duchêne, Molak, et al. 2014). However, similarly to partition finding software (see the “Partition models” subsection), it relies on a known phylogenetic tree as input. It is currently unclear how robust this method is to misspecifications of the true underlying tree.

#### General recommendations

We suggest the following practices to specify molecular clock priors:

1. Clock model: Relaxed uncorrelated clocks such as UCLN and IGR should be generally preferred over other types of clock models, despite autocorrelated relaxed clock models being biologically often more realistic, but uncorrelated relaxed clock models performing more robustly (Lepage et al. 2007; Linder et al. 2011). UCLN and IGR should be preferred over the UCE model, as the UCE model implicitly assumes the same variance as the mean. Alternatively, a mixture model over several uncorrelated and/or autocorrelated models can be applied with very little additional computational cost (Darlim and Höhna 2024).
2. Number of independent clocks: Out of practical reasons, we generally recommend using only one or two (mitochondrial + nuclear) independent molecular clocks. In cases where the extant tree topology is known with confidence, ClockstaR can be used to identify the optimal clock-partitioning strategy.

### Incomplete Lineage Sorting

The evolutionary history of independent or unlinked loci can be discordant. Specifically, under the assumption of the multispecies coalescent (MSC) process, lineages might not coalesce within their ancestral species branch if the population size is large and/or the duration of the branch is short (Maddison 1997; Rannala and Yang 2003; Rosenberg and Tao 2008). The phenomenon when lineages have not coalesced within their corresponding ancestral species tree branch is often referred to as *deep coalescence*, which can result into *Incomplete Lineage Sorting* (ILS). In phylogenomic studies, it is common practice to account for the discordance between species tree and gene tree by using an MSC model (Rannala and Yang 2003). For tip-dating studies, the MSC model has been recently extended to the FBD-MSC (Fossilized Birth–Death-MultiSpecies Coalescent) model (Ogilvie et al. 2022). However, almost no tip-dating study to date has used a model to account for gene tree–species tree discordance (Fig. 2f), such as the FBD-MSC, most likely due to computational challenges and increased difficulty of Markov chain Monte Carlo (MCMC) convergence, which are already very high without the additional complexity of gene tree–species tree discordance. Instead, the common approach is to concatenate loci and assume the same underlying topology.

#### Open questions

ILS is not the only possible cause for gene tree–species tree discordance. This phenomenon can also result from other biological processes (such as horizontal gene transfer, gene duplication and loss, and hybridization; Degnan and Rosenberg 2009) or from modeling and inference artifacts (Gatesy and Springer 2014; Springer and Gatesy 2016). Few studies have attempted to distinguish different biological and artifactual sources of gene tree–species tree discordance in empirical data sets (Morales-Briones et al. 2021; Smith et al. 2023; Höhna et al. 2025), and more work is needed in that direction. How ILS impacts tip-dated phylogenies remains unclear. Failing to account for ILS might result in older divergence times for most nodes, regardless of whether they are younger or older, but in shorter time durations for the branches directly affected by topological discordance (Leaché et al. 2014; Mendes and Hahn 2016; Martin et al. 2017; Carruthers et al. 2022; Ogilvie et al. 2022). Furthermore, ILS plays a strong role in species radiations and remains a challenging and active topic in method development (Mendes and Hahn 2018).

Several recent studies have found evidence of pervasive ILS at different phylogenetic levels (e.g., Feng et al. 2022; Rivas-González et al. 2023; Song et al. 2023). However, the methods used in these studies might be biased toward detecting ILS *a priori* (Stiller et al. 2024). A thorough investigation of ILS in a tip-dating framework is needed to establish whether tip-dating analyses that do not account for ILS are systematically biased, and whether these biases concern younger (interspecific) divergences, older (between major clades) divergences, or are pervasive at every phylogenetic level. Moreover, as most tip-dating studies focus on a subset of loci instead of full genomic data, these loci could be chosen so that gene tree–species tree discordance is minimized (see, e.g., Mongiardino Koch 2021; Höhna et al. 2025). Specifically, in phylogenomics it is important to choose a trade-off for the molecular loci between being long enough to result into reliable gene tree estimate but not violating the assumption of no recombination within a locus (Mendes and Hahn 2016; Springer and Gatesy 2018; Mendes et al. 2019). These debates and practices in phylogenomics have yet to be explored for tip-dating studies with usually much fewer loci. Finally, ILS is not restricted to molecular sequence evolution, but to discrete and continuous character evolution as well with so far little explored consequences (Mendes et al. 2018; Parins-Fukuchi et al. 2021).

#### General recommendations

We suggest the following practices for gene tree discordance:

1. Before running a tip-dating analysis, it is good practice to estimate unrooted topologies for each individual gene or locus in the molecular data set.
2. If these topologies (gene trees) are strongly discordant—that is, different genes provide strong support for different topological relationships—then using the FBD-MSC prior (or other ways to account for gene tree–species tree discordance) might be necessary. Alternatively, filtering genes based on their phylogenetic signal or discordance can be a useful tool to overcome this discordance. However, this approach has some degree of circularity and should be used with caution, as subsampling of genes with high phylogenetic signal might result in systematically shorter terminal branches and longer internal ones (Molloy and Warnow 2018; Mongiardino Koch 2021; Mongiardino Koch and Milla Carmona 2024).
3. If gene trees for selected loci are concordant or only moderately discordant (i.e., if all clades with high posterior probabilities are shared by all gene trees, and all topological discordances between them are due to poorly supported clades), a model where all loci evolve under the same species topology, as it is usually the case in tip-dating analyses, should be sufficient.

## Morphological Evolution

Before the era of molecular phylogenetics, morphology was the exclusive information to infer relationships among organisms. Morphological characters play a crucial role in tip-dating analyses, because they provide the only connection between extant and extinct taxa, with the exception of rare taxa for which ancient molecular sequences are available (e.g., the giant deer [Lister et al. 2005] or arctic rhinoceros [Paterson et al. 2024]). The often fragmentary nature and incomplete character suite of fossils constitutes a challenge for incorporating sufficient character information in order to resolve their systematic position (Sansom et al. 2010; Sansom and Wills 2013; Zhang et al. 2016; Luo et al. 2020; O’Reilly and Donoghue 2021; Spasojevic et al. 2021). Further, divergence times may be overestimated when using highly incomplete fossils, because their phylogenetic position cannot be resolved accurately and branches may appear artificially long (Ronquist et al. 2016; Arcila and Tyler 2017; Kealy and Beck 2017; Spasojevic et al. 2021; Luo et al. 2023). However, it has been shown that incorporating morphological characters of fossil and living species into tree reconstructions allows for a better understanding of systematic relationships and evolutionary processes (Poe and Wiens 2000; Wiens 2000, 2003, 2004, 2006; Simões and Pierce 2021).

There are two types of morphological characters: discrete (qualitative) characters and continuous (quantitative) characters; the latter can derive from linear measurements or landmark coordinates from 2D or 3D geometric morphometrics. Although only discrete characters have traditionally been used in phylogenetic analyses, recent approaches worked on integrating continuous characters into phylogenetic inference (Parins-Fukuchi 2018a, 2018b; Álvarez-Carretero et al. 2019; Zhang, Drummond, et al. 2023). Both discrete and continuous morphological data can potentially be combined in a total-evidence tip-dating analysis (e.g., Zhang, Drummond, et al. 2023).

Finding and developing evolutionary models for morphological characters is an extremely challenging task and more research needs to be done to improve modeling discrete morphological evolution (Harrison and Larsson 2015; Wright 2019; Simões and Pierce 2021; Casali et al. 2023; Mulvey, May, et al. 2025). We discuss the properties of the two types of morphological characters, potential partitioning schemes and how to model morphological evolution with a specific focus on tip-dating studies.

### Discrete Characters

Discrete morphological characters have been used to reconstruct evolutionary relationships for more than one and a half centuries (Darwin 1859). Morphological data have evident advantages, for example, availability for extinct organisms, but also come with challenges, for example, amount of available data and objective scoring across researchers. We highlight several important features of discrete morphological characters that should be considered in study design and model choice, which include the following:

1. Explicit and detailed character and character state descriptions as well as clear delimitation of character states of discrete morphological data are key for reducing subjectivity and allow for objective and reproducible scoring (Sereno 2007).
2. Character state changes of most characters, except for presence/absence or meristic characters, likely represent a continuum (not discrete categories as they are scored), of which not all states will be observed throughout the evolutionary history of an organism (Gentry 2000; Wright 2019).
3. Character states do not have the same meaning between characters, that is, character state “1” in character X has a different meaning from state “1” in character Y and so on, whereas in molecular data an “A” is always adenine across all sites.
4. Each morphological character state identifies a completely different biological entity between characters. In molecular data, state changes have the same properties across the data set (e.g., a transversion from A to T will always imply a mutation of a single nucleotide base from an adenosine to a thymine). Conversely, in morphology one character state change in character X may require one or few genetic mutations (or none at all if the character is environmentally induced), whereas the same state change in character Y may require a large amount of genetic changes (Wright 2019).

These features make model development for morphological character evolution extremely challenging, and prevent the cross-application of complex molecular substitution models (such as GTR) to morphological data sets. The standard solution has been to apply the Mk model (Lewis 2001), which is a generalized Jukes–Cantor model (Jukes and Cantor 1969), and thus, one of the simplest models assuming equal evolutionary rates and equilibrium state frequencies. The simplicity of this model stands in contrast to the higher variability of discrete morphological characters compared with molecular data. Therefore, the assumptions of the simplistic Markov *k* (Mk) model can be rather unrealistic (see below), although recent work has not found model violation across a set of data sets when applying the Mk model (Mulvey, May, et al. 2025). Further challenges in morphological data are potential character dependencies such as between-character correlation, presence of parallel evolution, and/or convergence, which underlie discrete morphological data to mostly unknown extents (Harrison and Larsson 2015; Goloboff et al. 2019; Zhang, Drummond, et al. 2023).

Usually, morphological data in Bayesian phylogenetics have been incorporated as discrete characters, which is probably due to method availability and development (Lewis 2001; Nylander et al. 2004; Ronquist et al. 2012; Heath et al. 2014; Wright and Hillis 2014; Harrison and Larsson 2015; Wright et al. 2016; Wright 2019). Each character can have two (binary character) or multiple (multistate character) states with a diagnostic morphology. Characters may be scored with an intended polarity, that is, one state is hypothesized to be ancestral or plesiomorphic, whereas another state is hypothesized to be derived or apomorphic (Watrous and Wheeler 1981; De Queiroz 1985; Stevens 1991; Sereno 2007). Multistate characters may be (directionally) ordered, for example, state “2” can only be gained after development of state “0” and then “1”; therefore, state changes can only occur in a specific order (Swofford and Olsen 1990; Swofford and Maddison 1992; Slowinski 1993).

Assuming unordered characters, a morphological character is phylogenetically (or parsimony) uninformative if its state is constant or invariant, that is, scored with the same number throughout all taxa, or differs only in one Operational Taxonomic Unit (OTU) per alternative state. A character is informative if its states vary among the taxon set and are shared by at least two taxa (Swofford and Maddison 1992). Thus, ideally, characters are variable between OTUs, but not within OTUs. The main challenge for invariant characters is, that it is not possible to know which structure could be a character, if it is not variable across different taxa. Thus, besides the benefit of more phylogenetic information, most studies aim to include only phylogenetically informative characters, which must be modeled accordingly (see below).

#### Models of morphological character evolution

**Figure 6.**
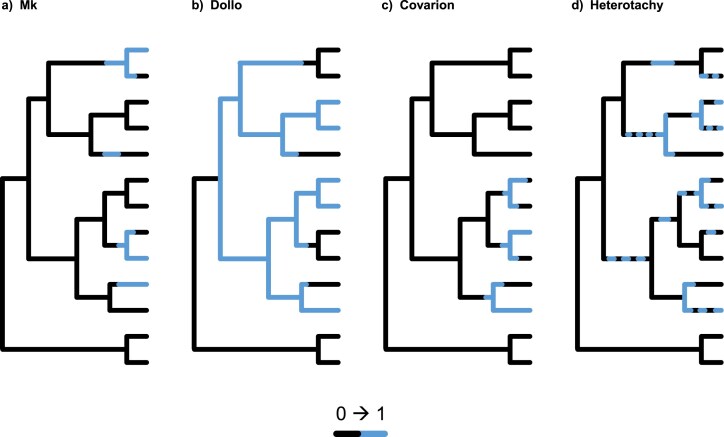
Different models for character evolution: (a) the traditional Mk model (Lewis 2001), (b) Dollo’s model, which is similar to the Mk model, but does not allow for regaining characters once they are lost, (c) the Covarion model, and (d) the heterotachy model. The latter two implying more relaxed assumptions on morphological evolution.

*Mk model*: The most commonly used transition model for discrete characters is the Mk model (Supplementary Fig. S4; Lewis 2001). There are five basic assumptions within the Mk model (Fig. 6): (i) characters are in one of the *k* discrete states, (ii) characters evolve independently of each other, (iii) character state changes are independent from the character history (Markov property), (iv) character state changes are instantaneous along branches, and (v) character states are neither ancestral nor derived (no directionality) (Wright et al. 2016). Further, the standard Mk model assumes that transitions between any character occur at the same transition rate $\mu$, and that the stationary frequencies of the Mk model are all 1/*k* for each character. For binary characters, the Mk model (or M2 because $k=2$) is represented by the transition rate matrix


(3) \begin{eqnarray*}
Q_{2} = {\begin{pmatrix}* &\quad \mu \\
\mu &\quad * \end{pmatrix}} \mbox{ .}
\end{eqnarray*}

In the general case for a multistate character with *k* states, the transition rate matrix is defined as


(4) \begin{eqnarray*}
Q_{k} = {\begin{pmatrix}
* &\quad \frac{\mu }{k-1} &\quad \cdots &\quad \frac{\mu }{k-1} &\quad \frac{\mu }{k-1} \\
\frac{\mu }{k-1} &\quad * &\quad \cdots &\quad \frac{\mu }{k-1} &\quad \frac{\mu }{k-1} \\
\vdots &\quad &\quad \ddots &\quad &\quad \vdots \\
\frac{\mu }{k-1} &\quad \frac{\mu }{k-1} &\quad \cdots &\quad * &\quad \frac{\mu }{k-1}\\
\frac{\mu }{k-1} &\quad \frac{\mu }{k-1} &\quad \cdots &\quad \frac{\mu }{k-1} &\quad * \end{pmatrix}} \mbox{ .}
\end{eqnarray*}

Equal transition rates and equal stationary frequencies might be unrealistic for most morphological characters (Klopfstein et al. 2015; Wright et al. 2016; Pyron 2017). The symmetry of the Mk model means that the rate of character state change to another is equal to its reversal, which in reality might be true for only a few characters. The Mk model was designed in this way so that any relabeling of the states of any character does not change the likelihood (in other words, swapping “0”s with “1”s for any number of characters results in the same likelihood). This property accounts for the arbitrary character state labeling in morphological data, that is, the character state “0” has a different meaning across all characters (Lewis 2001). However, it has been shown that simplistic morphological substitution models such as the Mk model used for morphological data in probabilistic approaches, particularly Bayesian inference, still result in more accurate topologies than inferred under maximum parsimony (O’Reilly et al. 2016, 2018; Keating et al. 2020; Simões, Vernygora, et al. 2020; Vernygora et al. 2020).

In the last decade, there have been several approaches to improve the Mk model concerning heterogeneity of evolutionary rates, nonstationary frequencies, and alternatives to the character rate variation (Harrison and Larsson 2015; Klopfstein et al. 2015; Wright et al. 2016; Pyron 2017; Capobianco and Höhna 2025). Major extensions of the Mk model are addressed in the following.

*Unequal stationary frequencies*: The frequencies of states (e.g., “0”s and “1”s) can deviate from the assumption of being equal. In molecular phylogenetics, unequal stationary frequencies are modeled by the Felsenstein (1981) substitution model (F81). The F81 model for a binary character gives the transition rate matrix by


(5) \begin{eqnarray*}
Q_{\pi } = {\begin{pmatrix}* & \pi _1 \\
\pi _0 & * \end{pmatrix}},
\end{eqnarray*}

where $\pi _0$ and $\pi _1$ are the frequencies of “0”s and “1”s, respectively. Very importantly, applying this F81 across an entire morphological data set assumes shared frequencies “0”s and “1”s across characters; thus, “0”s and “1”s should have the same meaning between characters (Lewis 2001; Klopfstein et al. 2015). If characters arbitrarily used the labels “0”s and “1”s and a reversal of labels would be equally valid for some characters, then the F81 model should not be applied without careful consideration.

One solution to keep symmetry and avoid different likelihood based on character relabeling is to apply symmetric mixture models where each character evolves under a mixture of rates, for example, $\mathbf {\pi } = \lbrace [\pi ^1_0,\pi ^1_1], [\pi ^2_0,\pi ^2_1], [1-\pi ^1_0,1-\pi ^1_1],[1-\pi ^2_0,1-\pi ^2_1]\rbrace$ (Wright et al. 2016). Note that $\pi _1=1-\pi _0$, as these represent frequencies. For multistate characters, such extensions might be possible but have not been developed while keeping the relabeling property (e.g., Klopfstein et al. 2015; Pyron 2017). Assigning each character its own set of stationary frequencies would keep the relabeling property and could be an alternative way forward, but would complicate the correct modeling of ascertainment bias correction (Capobianco and Höhna 2025).

*Ordered Q-matrix*: Many discrete morphological data sets contain ordered characters (Fig. 2). Evolution of an ordered character is assumed to follow only through consecutive character states. For example, a five-state ordered model is represented by the rate matrix


(6) \begin{eqnarray*}
Q_{\mathrm{ord}} = {\begin{pmatrix}
* &\quad \alpha &\quad 0 &\quad 0 &\quad 0 \\
\beta &\quad * &\quad \alpha &\quad 0 &\quad 0 \\
0 &\quad \beta &\quad * &\quad \alpha &\quad 0 \\
0 &\quad 0 &\quad \beta &\quad * &\quad \alpha \\
0 &\quad 0 &\quad 0 &\quad \beta &\quad * \end{pmatrix}},
\end{eqnarray*}

where $\alpha$ is the rate of gain (or increase) and $\beta$ is the rate of loss (decrease). Most often, the rates of gain and loss are assumed to be equal ($\alpha = \beta$). The ordered character transition model can be used for any multistate character, that is, there is no limitation on the maximum state. However, both the minimum state (usually “0”) and the maximum state need to be defined.

*Ascertainment bias (Mkv and Mkp)*: For morphological data, invariant characters and those with only one change in terminal branches of the tree are usually not collected; this is referred to as the *ascertainment bias* and needs to be corrected (Lewis 2001; Wright 2019; Capobianco and Höhna 2025). The simplest correction is to assume that all invariant characters are removed from the data set, as in the Mkv model. Note that the assumption clearly specifies that invariant characters (i.e., where all sampled taxa have the same character state) are removed and not only invariable characters (i.e., characters that cannot vary). For a binary character, the possible invariant observations are all taxa having a “0” state or all taxa having a “1” state. For multiple characters, this can easily be extended and there are exactly *k* such patterns for a character with *k* states. Then, the probability of each observed character is conditioned on not being invariant (i.e., divided by the probability of one minus the probability of the invariant patterns). Failing to account for the ascertainment bias correction can have drastic impact on branch length estimation (Lewis 2001).

The second ascertainment bias correction is when only parsimony informative characters are considered, as in the Mkp model (Allman et al. 2010; Ronquist et al. 2012; Matzke and Irmis 2018). For a binary character, parsimony uninformative patterns are the invariant patterns and patterns where only one taxon differs in characters state from all other taxa. Hence, there are in total $2+2n$ parsimony non-informative patterns, where *n* is the number of taxa. Again, the conditioning is done by dividing each observed character pattern by the probability of all excluded patterns. Because the number of parsimony non-informative patterns grows very rapidly for more than binary characters (Matzke and Irmis 2018), this correction is not applicable/feasible for multistate characters.

As morphological character matrices are often reused from other studies, it is likely to occur that a character that was parsimony informative (or not invariant) for a larger clade is not parsimony informative (or invariant) for a subclade. Software (e.g., RevBayes, Höhna et al. 2016) exclude these characters from an analysis if the specified ascertainment bias correction is violated.

Finally, the treatment of missing data complicates ascertainment bias corrections. For example, an observed character could be considered invariant when all but two taxa are scored as “0” and the remaining two taxa are scored as “?”. The likelihood for such a character can be computed, and thus it can be included in the analyses, but one could not easily condition on such characters being excluded (ascertainment bias correction) because the number of possible patterns is too large.

*Among-character rate variation*: Similar to molecular characters, morphological characters may vary in their rate of evolution. On one hand, morphological characters are often chosen to represent synapomorphies, that is, displaying only one shared change across the phylogeny, and to avoid homoplasy. These characters would not show any evolutionary rate variation. On the other hand, characters can also be defined to resolve the phylogeny on different levels (e.g., higher taxonomic level vs. lower taxonomic level) where some of these characters evolve very slowly and other evolve faster.

Several recent studies showed that phylogenetic inference was improved when modeling ACRV in morphological matrices (Rosa et al. 2019; Gonçalves et al. 2022). To accommodate ACRV, the same approaches as for molecular data are used (Yang 1994), primarily owing to the availability of existing software and methods. The distribution of character rates is often modeled by a gamma distribution. However, instead of drawing the character rates from the full gamma distribution, the common approach is to compute the quantiles of the gamma distribution and use these as rate categories, that is, discretizing the distribution. Then, each character has the same prior probability to evolve under any of the rate categories. Finally, the probability of observing the character is computed by summing overall rate categories.

ACRV models can be—and are often—combined with the aforementioned ascertainment bias correction for not observing invariant characters. However, when this is done, the correction can be calculated in two distinct ways: marginal ascertainment bias (mMkv model) and joint ascertainment bias (jMkv model) (Capobianco and Höhna 2025). Following Capobianco and Höhna (2025), we recommend to always use the mMkv model when applying ACRV to discrete morphological data.

In contrast to models of molecular evolution, there has been some work exploring the use of probability distributions alternative to the gamma distribution, and the number of rate categories (Wagner 2012; Harrison and Larsson 2015; Wright and Wynd 2024) (Supplementary Table S1). For example, a lognormal distribution instead of a gamma distribution and eight instead of four discrete categories have been explored (Harrison and Larsson 2015). A lognormal distribution might be more appropriate to model ACRV when there are only few morphological characters that evolve much faster than most characters in the data set (Wagner 2012; Harrison and Larsson 2015). However, the impact of different ACRV models on topological estimates has been barely explored, and it is unknown whether ACRV model choice can affect divergence time estimates and tip-dating analyses. Thus, research on modeling ACRV in morphological data sets is still in its early stages, and more exploration is needed to shed light into appropriate models for ACRV in morphological data sets.

*Additional models of morphological evolution*: Several additional models of morphological evolution have been described in the literature but have not been applied in tip-dating studies to date (see Fig. 6). Here, we briefly discuss some of these additional models to motivate further discussion in the community and development of better and more adequate morphological character evolution models.

Complex characters, for example, the ability of flight in birds (Phillips et al. 2010), are often assumed to be irreversible once lost (Dollo’s law, Dollo 1893). In likelihood-based phylogenetics, this irreversibility can be modeled. For example, one possibility implements irreversible evolution akin to Dollo’s law by modeling the number of newly evolved complex traits together with their loss (Alekseyenko et al. 2008; Nicholls and Gray 2008). Thus, the model consists of two parts: the number of complex traits (which are assumed to appear only once) and the independent loss of the trait at one or multiple lineages. The challenge of this model is that the origin of complex traits is not independent among lineages and standard algorithms such as Felsenstein’s pruning algorithm (Felsenstein 1981) cannot be used.

An alternative model for irreversible evolution of complex characters is to expand a binary character (e.g., “absent” and “present”) into a corresponding three-state character (e.g., “absent,” “present,” and “lost”). Since we cannot know *a priori* for any taxa without the complex trait if it was lost or never had been gained, the character is simply coded as ambiguous between the two. Then, a transition rate matrix for this three-state character can be defined as


(7) \begin{eqnarray*}
Q_{\mathrm{Dollo}} = {\begin{pmatrix}
* &\quad \lambda &\quad 0 \\
0 &\quad * &\quad \mu \\
0 &\quad 0 &\quad * \\
\end{pmatrix}},
\end{eqnarray*}

where $\lambda$ is the rate of gain of this complex character and $\mu$ is the rate of loss. The third row, representing the “lost” state, is a sink state and the process will never leave the state once entered. Contrary to the irreversible character evolution model of Alekseyenko et al. (2008), this model allows for multiple gains of the complex character.

Another type of morphological character evolution, the *covarion* model, focuses on the speed of evolution among different lineages. An unobserved character (or external factor such as habitat) influences whether the focal character is slow- or fast-evolving (e.g., Beaulieu et al. 2013). For example, we can expand the binary Mk model to incorporate fast ($\mu _f$) and slow ($\mu _s$) evolution as


(8) \begin{eqnarray*}
Q_{\mathrm{Cov}} = {\begin{pmatrix}
* &\quad \mu _s &\quad \delta &\quad 0\\
\mu _s &\quad * &\quad 0 &\quad \delta \\
\delta &\quad 0 &\quad * &\quad \mu _f\\
0 &\quad \delta &\quad \mu _f &\quad * \end{pmatrix}},
\end{eqnarray*}

where $\delta$ is the rate of switching between the slow and fast categories. We can even assume that the slow rate $\mu _s=0$ so that no evolution happens (i.e., the character is switched off). Such models have been explored for molecular phylogenetics (Tuffley and Steel 1998; Penny et al. 2001; Galtier 2001) but not for morphological phylogenetic inference (but see Beaulieu et al. 2013, for a comparative study investigating the evolution of the trait itself).

A less mechanistic and more phenomenological model than the covarion models is a *heterotachy* model (Philippe and Lopez 2001; Lopez et al. 2002) focusing on the phenomenon that per character rates may vary along lineages (Fig. 6d). A heterotachy model assumes that each character can have its own set of branch lengths; thus, the evolutionary speed of different characters varies among lineages. The No Common Mechanism model—which corresponds to the maximum parsimony method when implemented in a maximum likelihood framework—is a heterotachy model where the number of parameters grows faster than the number of characters added to the data matrix (Tuffley and Steel 1997; Huelsenbeck, Ane, et al. 2008). Less extreme versions of heterotachy, where not each character has its own branch lengths but these are shared among some characters or where variation in branch lengths is governed by a prior distribution, are computationally easier but less explored for phylogenetic inference (Pagel and Meade 2008).

Finally, *character dependency* is a long-known challenge in phylogenetic inference of morphological characters. Whether or not and to what extent morphological characters are correlated with each other and how much convergence is involved is one of the biggest challenges. Note that standard phylogenetic models of discrete characters (of both molecular and morphological data) assume that each site evolves independently. Thus, modeling character dependency provides a general challenge.

One approach to model character dependencies is via *latent liability* models (Cybis et al. 2015; Felsenstein 2005). In the latent liability model (or threshold model), the underlying character is assumed to be continuous but the observed character is discrete (Felsenstein 2005). It is then possible to apply any standard continuous trait evolution model, for example, Brownian motion or Ornstein–Uhlenbeck (OU) process (see the next section), even modeling covariation using the multivariate continuous character evolution models (Cybis et al. 2015).

Another alternative to model character dependency of two discrete morphological characters is by combining the two characters into a single one (Pagel 1994; Tarasov 2019). For example, if the first binary character has states “0” and “1” whereas a second binary character has states “A” and “B,” the combined character would have four states: “0A,” “0B,” “1A,” and “1B.” The transition rate matrix of the combined character can be written as


(9) \begin{eqnarray*}
Q_{\mathrm{Corr}} = {\begin{pmatrix}
* &\quad \mu _{01A} &\quad \mu _{AB0} &\quad 0\\
\mu _{10A} &\quad * &\quad 0 &\quad \mu _{AB1} \\
\mu _{BA0} &\quad 0 &\quad * &\quad \mu _{01B} \\
0 &\quad \mu _{BA1} &\quad \mu _{10B} &\quad * \end{pmatrix}},
\end{eqnarray*}

where the indices of $\mu$ indicate first the transition and then the conditional state; for example, $\mu _{01A}$ means a transition in the first character from “0” to “1” while being in state “A” of the second character. Note that simultaneous transition of both characters is prohibited because transitions are assumed to occur instantaneously. The transition rate can be arbitrarily restricted and assuming $\mu _{01A} = \mu _{01B}$, $\mu _{10A} = \mu _{10B}$, $\mu _{AB0} = \mu _{AB1}$, and $\mu _{BA0} = \mu _{BA1}$ represents the uncorrelated model. This type of character correlation models can also be used to properly account for inapplicable character states. If the second character cannot be observed for both states of the first character (e.g., tail color can only be observed if there is a tail), then the inapplicable state of this second character can be scored using ambiguity (Tarasov 2019).

#### Open questions

Constructing a data set of only parsimony informative characters has one major drawback especially for tip-dating studies: there is no information in the data set about the length of terminal branches as most transitions on these branches are excluded. It is therefore prudent to rethink the common practice of selecting characters based on their phylogenetic information. As invariant characters are unlikely to be included in their correct proportion to variant characters, this naturally needs to be corrected for, and the common exclusion of invariant characters (e.g., Mkv) might be the safest option. Nevertheless, since there is no correction for weight between variant but parsimony uninformative character and parsimony informative characters, researchers should strive to include all variable characters to obtain realistic estimates of branch lengths and thus divergence times (Lee and Palci 2015; Matzke and Irmis 2018).

Due to its simplistic assumptions, the Mk model is likely not the most adequate for modeling morphological evolution of empirical data sets (but see Mulvey, May, et al. 2025). Future studies should investigate how inadequate models affect phylogenetic inference and which extensions to the Mk model should be implemented to compensate for potential shortcomings. We listed several extensions that have been described in the literature but have not (yet) been tested in Bayesian tip-dating studies. Several macroevolutionary studies exploring the mode of discrete character evolution rejected the Mk model (i.e., equal rates Markov model), and supported unequal rates or irreversible models. Thus, models used for inferring phylogenies based on discrete morphological data differ from models used for investigating discrete character evolution on a given phylogeny: a discrepancy that needs to be addressed in future research.

#### General recommendations

We suggest the following practices for scoring and modeling discrete morphological characters:

1. Character definition: Objective character and character state definitions are essential for comprehensible documentation of discrete morphological data.
2. Character inclusion: All variable characters, including parsimony non-informative characters, should be included to obtain robust estimates of branch lengths and divergence times.
3. Character modeling: Until more Mk model extensions or other models for morphological evolution are empirically tested and established, we recommend applying the Mk model with the marginal ascertainment bias correction (Lewis 2001; Capobianco and Höhna 2025) and a model of ACRV, with a discrete gamma or lognormal distribution (e.g., the mMkv+$\Gamma$ model, Capobianco and Höhna 2025). If additional information about characters is available, for example, whether these character are ordered, then this information should be included in the model appropriately. Similarly, dependent characters should be coded and modeled appropriately (Brazeau 2011; Tarasov 2019). With development of biologically more realistic models of discrete character evolution, our recommendations will need revision; however, some recent work indicates that even the simple Mk model may be adequate in some cases (Mulvey, May, et al. 2025).

### Continuous Characters

Continuous characters add a third independent component for phylogenetic inference (in addition to molecular and discrete morphological data, Fig. 1) and enable incorporating more data and taxa—especially concerning fossils—into phylogenetic analyses (Wright et al. 2016; Wright 2019; Zhang, Drummond, et al. 2023). Despite providing a rich source of morphological data, continuous characters have rarely been used for phylogenetic inference (Polly 2003; Smith and Hendricks 2013; Catalano and Torres 2017; Parins-Fukuchi, 2018a, 2018b; Álvarez-Carretero et al. 2019; Ascarrunz et al. 2019; Zhang, Drummond, et al. 2023). Traditionally, only discrete characters have been used in tip-dating analyses, and incorporating continuous data would be the natural next step to expand those analyses and bring a new perspective to investigate phylogenetic relationships and evolutionary processes (Álvarez-Carretero et al. 2019; Zhang, Drummond, et al. 2023). When continuous characters have been included in phylogenetic studies so far, most of the times they have been transformed into characters with a discrete number of states. This is not unproblematic, because it introduces an unnecessary subjectivity to otherwise objective measurements. Modeling originally quantitative traits as discrete characters poses an additional challenge (Wiens 2001).

Advantages associated with the use of continuous characters include that they may contain more information than discrete characters because they are variable across all species (Parins-Fukuchi 2018b). Thus, inferring phylogenetic trees with continuous data could come with lower topological error compared to analyses from the same number of discrete characters (Parins-Fukuchi 2018b). Further, because continuous characters are variable, there is no need for an ascertainment bias correction as there is for discrete characters.

Another advantage concerns the long history of statistical models for continuous character evolution, which have been developed over more than 50 years to capture a variety of evolutionary processes such as drift, stabilizing selection, directional evolution, early burst, and saltational evolution (Felsenstein 1973, 1988; Hansen 1997; Blomberg et al. 2003; Landis et al. 2013; Uyeda and Harmon 2014).

Potential limitations on the use of continuous characters include high levels of character correlation and intraspecific variation (but see Álvarez-Carretero et al. 2019; Zhang, Drummond, et al. 2023 for possible ways to account for these). Additionally, although continuous characters can be measured objectively rather than being discretized more or less subjectively, the choice of which ones to include in a phylogenetic analysis remains subjective, as for discrete characters.

#### A priori character treatment

Continuous character data can come from two major sources: landmark-based 2D or 3D geometric morphometrics (shape data), or linear measurements. Both kinds of data require some processing to normalize the characters before their analysis. For continuous data derived from 2D or 3D geometric morphometrics landmark analyses, the data processing includes a step called Procrustes superimposition, in which landmarks are translated, rotated, and scaled in order to align them and thus standardize them (Bookstein et al. 1985; Zelditch et al. 2012; Álvarez-Carretero et al. 2019; see also Cardini and Marco 2022) for common pitfalls and recommendations in analyzing Procrustes shape data).

For metric data derived from linear measurements, several options are available. Data could be log-transformed, transformed by a linear regression analysis proceeding with the residuals, or each measurement could be divided by a value that is representative of body size, for example, body mass or total skull length (Logan 2011). Ratios between two linear measurements should always be log-transformed (Mongiardino Koch et al. 2015).

#### Brownian motion

The simplest way of modeling evolution of quantitative characters is by Brownian motion, which is also often referred to as the random walk (Felsenstein 1973; Butler and King 2004; Parins-Fukuchi 2018b). The Brownian motion model contains only a single parameter: the evolutionary rate $\sigma$ determining the amount of drift over time. It is assumed that the character at the end of a branch ($X_D$: descendant value) is normally distributed centered around the character value at the beginning of the branch ($X_A$: ancestral value) with standard deviation $\sigma$ times the square root of the length of the branch *t*,


(10) \begin{eqnarray*}
X_D \sim \mathrm{Normal}(X_A,\sigma *\sqrt{t}).
\end{eqnarray*}

The Brownian motion model assumes that characters evolve purely under drift without any constraints, for example, without any boundary or optima to which the character is attracted to. This unconstrained drift assumption leads to an increasing variance the longer the process continues, which can be biologically unrealistic. Note that classical phylogenetic Brownian motion models with trend are not identifiable, but such models are identifiable when used for phylogenies with extinct samples (Silvestro et al. 2019).

#### Ornstein–Uhlenbeck

Alternatively, continuous character evolution has been modeled by an OU process (Felsenstein 1988; Hansen 1997; Parins-Fukuchi 2018b), where the character is attracted to an optimum value $\theta$ with attraction rate $\alpha$ (or selective force), given by


(11) \begin{eqnarray*}
X_D \sim \mathrm{Normal}(X_A * e^{-\alpha *t} + (1- e^{-\alpha *t})*\theta ,\sigma *\sqrt{t}).
\end{eqnarray*}

The appealing characteristic of the OU process is that it is bounded and models evolution under selection toward a fitness optimum. However, there are several disadvantages intrinsic to the OU process. First, selection toward the optimum erases phylogenetic signal. The speed of loss of phylogenetic signal can be computed by the phylogenetic half-life $t_{1/2}=\frac{\ln (2)}{\alpha }$. Second, parameterizing the OU process is challenging because of interactions and identifiability of parameters (Ho and Ané 2014; Cooper et al. 2016; Cornuault 2022). Nevertheless, a well-specified OU process model has the ability to collapse to a Brownian motion (if the rate of attraction $\alpha = 0$) or a white noise process (if the phylogenetic half-life is much smaller than the tree age).

It is perhaps surprising that continuous characters have not been used more often in tip-dating studies (Fig. 2h). The reasons for this are various. Only recently few phylogenetic software (e.g., BEAST2 and RevBayes) implemented the multiple modeling components required to integrate continuous character evolution with time-calibrated phylogenetic inference. Other reasons may include: researchers being more familiar with models for discrete characters; availability of appropriate data sets; or study designs chosen to incorporate and score discrete morphological characters only (Lee and Palci 2015; Zhang, Drummond, et al. 2023). Additionally, although some studies have highlighted the potential of continuous morphological characters and their success in simulations and a few empirical case studies (e.g., Parins-Fukuchi 2018a, 2018b; Álvarez-Carretero et al. 2019; Zhang, Drummond, et al. 2023), others have shown poor performance of landmark data for phylogenetic inference (Catalano and Torres 2017) or outright questioned whether phylogenies can ever be estimated reliably from shape data, especially when phenotypic integration and stabilizing selection are involved (Varón-González et al. 2020). We advocate for a continued research effort to explore the inclusion of continuous characters in Bayesian phylogenetic inference, as progress in this field will allow to unlock a currently underutilized source of data for future tip-dating analyses.

#### Open questions

As continuous morphological characters have rarely been used in tip-dating studies, there are several modeling choices that should be explored in the future. First, the current approach to remove rate variation among characters is by normalizing the character so that all have the same variance (Álvarez-Carretero et al. 2019). However, as outlier values can heavily influence the variance, it is not known if this normalization truly removes ACRV. Second, partition models may play an important role and could be applied similar to discrete morphological character partitioning schemes, for example, by anatomical region. Third, OU or other more complicated models, for example, Levy-jump models, should be explored as alternatives for Brownian motion models beyond the simple applications, that is, with among-character correlations and within-species variation or measure errors (Bastide et al. 2018; Caetano and Harmon 2019; Álvarez-Carretero et al. 2019).

Empirical assessment of continuous characters for likelihood-based phylogenetic inference has mostly focused on shape data derived from 2D or 3D geometric morphometrics, with contrasting results (Catalano and Torres 2017; Parins-Fukuchi 2018a; Zhang, Drummond, et al. 2023). Conversely, little attention has been given to the use of linear measurements (but see Parins-Fukuchi 2018a). This is surprising because linear measurements and proportions are already routinely employed in phylogenetic inference as discrete characters with an arbitrary number of states (Thiele 1993; Wiens 2001). Further assessment of this type of continuous characters modeled under Brownian motion and OU processes in a tip-dating framework is required.

#### General recommendations

We suggest for modeling continuous characters:

1. Character treatment: As for discrete characters, transparent character documentation is essential and continuous data should be normalized prior to phylogenetic analysis.
2. Character selection: We encourage scoring of both continuous and discrete characters for as many taxa as possible, because an increased number of taxa with an overlap of discrete and continuous characters seem to increase precision of divergence time estimates (Zhang, Drummond, et al. 2023).
3. Modeling rate variation: We recommend modeling ACRV if characters are not normalized to have the same variance and thus the expected evolutionary rate (Álvarez-Carretero et al. 2019).
4. Modeling character correlation: Accounting for character correlation and intraspecific variation is more feasible for continuous character than for discrete morphological character and should be applied whenever possible (Álvarez-Carretero et al. 2019).
5. Modeling continuous character evolution: Where computationally feasible, we recommend making more use of the OU model, even though it has not yet been exhaustively explored in simulations, because it collapses to a BM model and is biologically more realistic. However, the BM model is currently more commonly applied due to its wider software availability (but see Parins-Fukuchi 2018b for an example using RevBayes) and seems to model most data sets well enough.

### Partitioning Approaches for Morphological Characters

Morphological data sets have received less attention regarding partitioning the data into subsets compared with molecular data sets. This might be partly due to fewer available model choices for morphological data sets and to the predominant use of parsimony inference, where data partitioning cannot be applied in the same way. Morphological data sets—similar to molecular data—are heterogeneous, including subsets, for example, individual characters or groups of characters, which evolve differently along the phylogeny (Clarke and Middleton 2008; Close et al. 2015; Rosa et al. 2019; Simões and Pierce 2021; Casali et al. 2022; Gonçalves et al. 2022).

**Figure 7.**
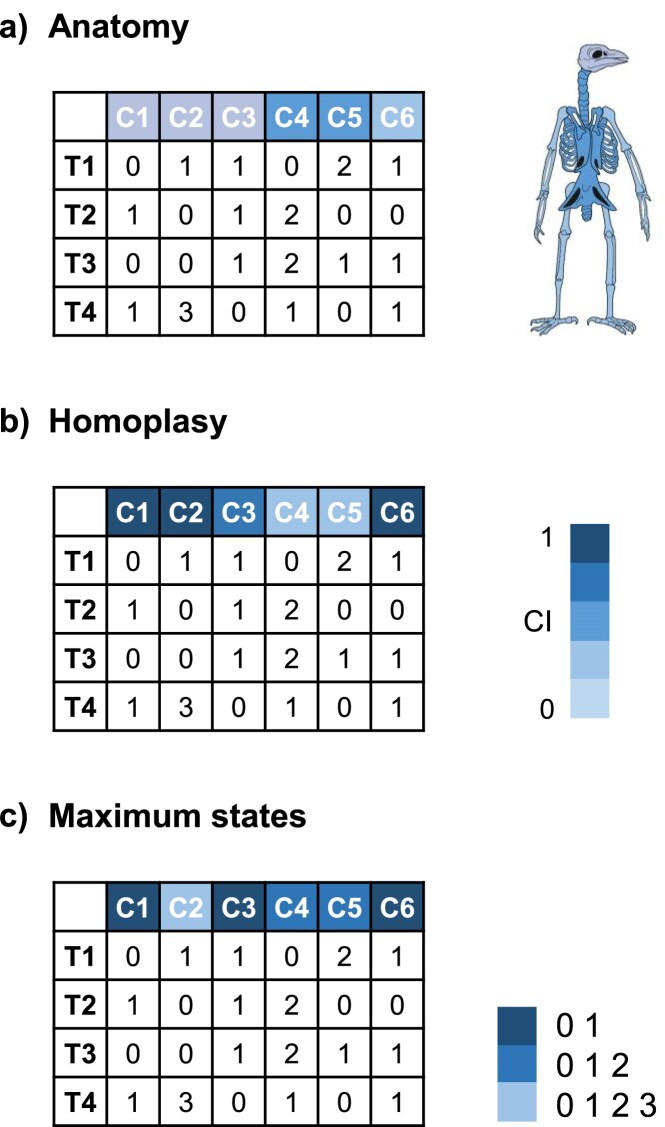
Partitioning morphological data: (a) traditional partitioning by anatomical region such as cranial or postcranial, (b) partitioning by homoplasy using the consistency index, and (c) a common partitioning scheme using the maximum number of character states as the partitioning criterion.

First, partitioning morphological data by anatomy (Fig. 7a) is a common and intuitive method, following the principle that anatomical or morphofunctional subregions, for example, cranial, dental, or postcranial, evolve at different rates (Clarke and Middleton 2008; Tarasov and Genier 2015; Porto et al. 2021). Partitioning by anatomical region with linked branch lengths has little influence on the precision and accuracy of the topologies, whereas models with unlinked branch lengths across partitions improve the precision but decrease the accuracy (Casali et al. 2023). Second, morphological data matrices can be partitioned by homoplasy, which has been shown to outperform other partitioning schemes (Fig. 7b) (Rosa et al. 2019; Casali et al. 2022). For each character the consistency index is calculated based on the most parsimonious tree and characters with a similar consistency index are grouped together. Using a phylogeny to calculate the consistency index might create circularity in the phylogenetic inference, which is a fundamental drawback of this method. Further, automatic partitioning of morphological characters for partitioned relaxed morphological clock analyses was introduced as an extension to investigate evolution in clock-based Bayesian framework and a step toward more similar partitioning procedure as in molecular phylogenetics (Simões and Pierce 2021). This method identifies the best partitioning scheme by calculating pairwise distances (Euclidean distance matrices) between characters and is followed by subsequent testing these schemes using clustering and ordination methods (Goswami and Polly 2010; Lanfear et al. 2017; Simões and Pierce 2021). Finally, morphological data matrices can be partitioned by number of possible character states (Gavryushkina et al. 2017; Khakurel et al. 2024). Because morphological data sets can include a combination of binary and multistate characters with varying state space (Supplementary Fig. S6), a common way to partition morphological data is by the maximum number of states observed per character (Fig. 7c; Supplementary Fig. S5; Khakurel et al. 2024). Thus, each partition can have a transition rate matrix *Q* of the adequate size. In other words, transition rates of binary characters can be modeled by a 2×2 *Q* matrix, those of three-state characters can be modeled by a 3×3 *Q* matrix, and so on. In fact, by default MrBayes (Ronquist et al. 2012) and BEAST (when using BEAUti) (Drummond et al. 2012) automatically partition morphological data by the maximum number of states per character. Partitions by state space usually share the same character rate (i.e., the size of the *Q* matrix varies, but the transition rate $\mu$ stays the same). Ultimately, all these partitioning schemes are not mutually exclusive and can be nested in various ways. For example, data subsets partitioned by anatomy can each have characters with different number of states that share the same rate but are partitioned by state space, so that their transitions can be modeled by a *Q* matrix of the appropriate size.

Once the morphological data matrix is partitioned into data subsets, the next step is to determine shared (i.e., linked) and independent (i.e., unlinked) parameters. Because the only applied transition model in tip-dating analyses is the Mk model, which only has one overall rate of transitions “$\mu$” that is directly linked to branch rates, the substitution model is the same among all partitions with the exception of different state spaces (*k* might change among partitions; Khakurel et al. 2024). Thus, the only parameters that can be unlinked among morphological data subsets are (1) the overall rate of evolution (scaling the tree), (2) the ACRV, and (3) the branch lengths. Several studies have indicated that the rate of evolution and/or independent branch lengths have the largest impact on model fit and sometimes lead to different tree inferences (Clarke and Middleton 2008; Rosa et al. 2019; Gonçalves et al. 2022; Casali et al. 2023). The challenge with applying independent branch lengths per data subset is that $2n-2$ (we assume rooted trees as we focus on tip-dating studies) additional parameters are required for each subset after the first one, where *n* is the number of tips. The prevalence of finding unequal branch lengths might indicate that a heterotachy model would be more adequate (see above).

#### Open questions

Partitioning morphological data sets has not received much attention except for the often implicit but overlooked division by maximum observed character state (for details of this approach, see Khakurel et al. 2024). The main impact of assuming an incorrect state space is branch lengths, which can have important implications in tip-dating studies for divergence time estimates (Khakurel et al. 2024; Mulvey, May, et al. 2025). The implications of partitioning morphological data sets have received little attention in the context of tip-dating studies, where morphology is the only data that inform branch lengths of extinct lineages and therefore calibrate the phylogeny (but see Lee 2016 for an empirical tip-dating example on linking vs. unlinking clock rates across morphological data subsets).

The major open question is what parameters, either the overall (partition) rate, the ACRV rate, or morphological relaxed clock rates, should be unlinked across partitioned subsets. Further, establishment of biologically more realistic transition models will result in more complex partitioning schemes for morphological data matrices, and thus will make well-considered partitioning important. Several data subsets with only a single character or few characters, for example, for multistate characters or ordered characters, will receive their own transition rate matrix but clearly do not have sufficient information to have other independent parameter, such as branch lengths.

#### General recommendations

Different taxonomic groups will need different hypotheses of partitioning; however, there is no guidance toward optimal or minimum subset size (Rosa et al. 2019). We suggest the following practices for morphological data partitioning:

1. We suggest that partitioning by maximum character state should be applied, although care should be taken as the observed state space might not correspond to the “true” biological state space (Khakurel et al. 2024).
2. Further partitioning by homoplasy index or rate of evolution might not be necessary if ACRV is modeled (Rosa et al. 2019; Casali et al. 2022; Gonçalves et al. 2022).
3. The main beneficial parameter when unlinked between morphological data subsets has been branch lengths (Clarke and Middleton 2008), which means for tip-dating analyses that different relaxed-clock models might be required per morphological data subset (Lee 2016).

### Morphological Clocks

Tip-dating studies using morphological data fundamentally rely on the assumption of a morphological clock. The simplest, albeit most unrealistic, assumption is a strict morphological clock. That is, each lineage evolves at the same overall morphological rate (though characters might evolve at different rates; see the “Among-character rate variation” subsection) and the number of morphological changes is proportional to time. The strict morphological clock assumption can be relaxed similar to molecular relaxed clocks (Thorne et al. 1998; Huelsenbeck et al. 2000; Drummond et al. 2006). Each branch can receive its own branch-specific clock rate that is drawn from some prior distribution (see Table 1). The variance in the branch-specific clock rates quantifies how similar the rates of morphological evolution are over time and among lineages. Assuming some type of morphological clock model, either strict or relaxed, is essential for a tip-dating analysis; otherwise, branch length and branch time are not identifiable (Donoghue and Yang 2016). Further, the morphological clock can be assumed to be either linked to or independent of the molecular clock.

The concept of morphological characters having varying evolutionary rates among lineages goes back to the Origin of Species (Darwin 1859). The presence of extremely slow and fast rates of phenotypic evolution, similarly to varying clock rates across lineages in molecular evolution (Drummond et al. 2006), has been shown in a plethora of studies (Haldane 1949; Simpson 1953, 1984; Gingerich 1983, 1993). Morphological characters evolved by adapting to different selective pressures between lineages. Therefore, heterogeneous evolutionary rates across lineages should be expected (Lee et al. 2014; Lee and Palci 2015; dos Reis et al. 2016; Donoghue and Yang 2016; Drummond and Stadler 2016; Goloboff et al. 2019).

Different morphological characters may show different variation in evolutionary rates among lineages, as can be seen by the support for unlinked branch lengths in morphological data partitions (Clarke and Middleton 2008). Thus, sufficiently large data sets will comprise groups of morphological characters that evolve at a shared rate, whereas other groups of characters evolve at different branch-specific clock rates, which makes the use of multiple morphological clocks preferable (O’Leary et al. 2013; Ni et al. 2013; Duchêne, Molak, et al. 2014; Lee 2016).

Using multiple clocks in tip-dating analyses, instead of a single clock, can affect topologies and divergence time estimates in tip-dating analyses, and potentially provide more detailed estimates of evolutionary patterns of distinct phenotypic regions (Beck and Lee 2014; O’Reilly et al. 2015; Simões, Caldwell, et al. 2020; Simões and Pierce 2021). However, due to the generally small size of morphological data sets (Fig. 2g) and consequent low amount of information present in each morphological partition, clock rate estimates for individual partitions might just recapitulate prior distributions, especially when these are co-estimated with the tree topology (Simões, Caldwell, et al. 2020). Hence, morphological clock partitioning might be a viable option for tip-dating phylogenetic inference only for data sets with a large number of characters.

Morphological character sampling can greatly impact clock estimates, and thus divergence times. Empirical morphological data sets tend to be unevenly sampled, with characters that vary only on terminal branches (autapomorphies) drastically undersampled compared with characters that vary on internal branches. Although this is certainly true for data sets that were originally designed for parsimony analyses—where autapomorphies are discarded *a priori*—even data sets that have not been designed for parsimony analyses can heavily undersample characters that vary on terminal branches (Lee 2016). This results in systematically shorter terminal branches, which can produce underestimated divergence times for shallower nodes and overestimated divergence times for deeper nodes (Lee 2016).

Overall, there has been no specific theoretical and methodological development of morphological clocks. Instead, the standard practice has been to adopt already existing approaches from molecular evolution (Table 1), which can be seen by the applied morphological clock models (Fig. 2d). Surprisingly, a high proportion of total-evidence tip-dating studies to date has employed one shared clock between molecular and morphological data (Supplementary Table S1). In these cases, partition rate multipliers can model differences in mean evolutionary rates between morphology and molecules. However, this is an appropriate way to model rates of morphological and molecular evolution only if we assume that their among-lineage rate variation is correlated (i.e., lineages that have higher rate of molecular evolution also have a proportionally higher rate of morphological evolution, and vice versa). Contrary to this assumption, molecular and morphological evolutionary rates are sometimes decoupled (Bromham et al. 2002; Halliday et al. 2019; Simões, Vernygora, et al. 2020; Asar et al. 2023).

#### Open questions

To our knowledge, there is no study that has demonstrated the clock-like behavior of morphological characters and proven their suitability for divergence time estimation (but see Lee et al. 2014). Therefore, there is still some skepticism whether morphological characters evolve in a clock-like manner, regardless of whether relaxed or not relaxed (discussed in Lee 2020). The plethora of studies on relaxed molecular clocks and their model development may be sufficient to model variation on morphological clocks; however, it should be investigated if there are clock-related features, which are unique to morphological evolution and should be modeled accordingly.

#### General recommendations

We suggest the following practices:

1. Morphological clock model: We recommend using a relaxed morphological clock that is unlinked (independent) from the relaxed molecular clock, either an IGR model or a UCLN model.
2. Morphological clock partitioning: For practical reasons, we recommend using only one morphological clock, with among-character heterogeneity rather modeled by ACRV, partition rate multipliers, and/or heterotachy. Multiple clocks might be reasonable to apply for large data sets (>300 characters), but careful comparison with effective prior distributions is advised.
3. Morphological character sampling: For accurate estimates of morphological clock rates, a thorough sampling of characters that vary only on terminal branches (autapomorphies) is fundamental. These characters are also essential for estimating divergence times of fossil lineages.

## Fossil Age Data and Lineage Evolution

The third main component of a tip-dating analysis is the lineage evolution model, that is, the prior distribution on the phylogeny together with tip ages (Fig. 1). Tip ages are derived from geological and paleontological data, which come with their own set of challenges. In fact, choosing the absolute age—in thousands or millions of years—of an extinct phylogenetic tip is not a trivial task. Further, different lineage evolution models can assume a different meaning of tip ages (age of sampled fossil specimens vs. age of sampled extinct species). We outline the different sources of age data for fossils and the uncertainties associated with them, and highlight how fossil ages are used in tip-dating analyses.

### Fossil Age Data

The fossil record provides crucial temporal and morphological information (Gandolfo et al. 2008). Fossils represent the source of a past diversity inaccessible by molecular data (Lee and Palci 2015). However, determining the age of a fossil in a phylogenetic framework can be contentious and has been debated in phylogenetics literature (Benton and Donoghue 2007; Bapst et al. 2016; Hopkins et al. 2018; Barido-Sottani et al. 2019).

Typically coming from sedimentary rocks, fossil specimens cannot be assigned to an exact age in most cases. In relative dating, a commonly used method, fossil ages are obtained based on the particular interval of geological time from the stratigraphic unit where the specimen was collected (Hopkins et al. 2018). Dates of these stratigraphic units are in turn based on the relative order of deposition between sedimentary layers and radiometric dated volcanic rocks (stratigraphy), and/or by comparison with similar strata that contains fossils with known ages (biostratigraphy) (Benton and Donoghue 2007; Parham et al. 2012; Landing et al. 2013; Hopkins et al. 2018). Alternatively, in absolute dating methods, radiometric dating (measure of isotopic decay) is able to date fossils directly. However, this approach is dependent on specific fossil types, besides being often limited to geologically younger fossils (Hopkins et al. 2018; Patzkowsky and Holland 2019). This in turn illustrates the imprecision associated with dating fossils (Hopkins et al. 2018).

### Specimen Stratigraphic Age Uncertainty and Taxon Stratigraphic Range

As discussed above, in most cases, the age of fossil specimens is assigned to a particular interval of geological time. This is defined as stratigraphic uncertainty of the age of a fossil specimen, in which the length of the geological time interval can vary significantly depending on the delimitation of the stratigraphic units (Benton and Donoghue 2007; Gandolfo et al. 2008; Ho and Phillips 2009; Hopkins et al. 2018; Parham et al. 2012). Usually, many fossil specimens are assigned to a single taxonomic unit (morphospecies) and the presence of such a taxon can sometimes span millions of years (Ho and Phillips 2009; Joyce et al. 2013). Therefore, a stratigraphic “range” is assigned to this fossil taxon (Parham et al. 2012; Barido-Sottani et al. 2019, 2023; Püschel et al. 2020). The range of a fossil taxon is determined by the oldest and the youngest specimens attributed to the taxon, which in turn represents the “first and last appearance dates” (Parham et al. 2012; Hopkins et al. 2018; Barido-Sottani et al. 2019). Stratigraphic age uncertainty is similarly applied to the “first and last” occurring specimens of a fossil taxon (see Hopkins et al. 2018 for a review). To avoid confusion, we recommend the usage of fossil or specimen age uncertainty when talking about stratigraphic age uncertainty, and stratigraphic “range” when talking about the duration of a morphospecies.

### Assigning Absolute Ages to Fossils in Tip-Dating

The following steps are necessary to derive the absolute age of a fossil specimen with its stratigraphic uncertainty for a tip-dating analysis:

1. Retrieve stratigraphic provenance (formation, group, etc.) of the fossil specimen by looking at publications describing or referring to that specimen, or at museum collection records associated with it.
2. Identify the age corresponding to the stratigraphic unit where the fossil comes from. This is often expressed in chronostratigraphic stages or series (e.g., Cisuralian, Cenomanian, Eocene, Messinian), but it can be expressed in biozones, magnetic polarity zones, or other timescale units. Usually this information is found associated with the stratigraphic provenance of the fossil specimen, but it might be worth to check the literature for recent stratigraphic assessments, especially in cases where the specimen description is several decades—or even centuries—old.
3. Convert the relevant timescale unit(s) into minimum and maximum absolute ages by consulting the latest version of the international geologic timescale (at the time of our writing: Gradstein et al. 2020).
4. In some cases, all the steps above could be replaced by directly extracting age data from large databases of fossil occurrences such as the Paleobiology Database (Dillon et al. 2023). However, the data quality and coverage of these databases is quite heterogeneous, varying with factors such as taxonomic group, geographic region, and geologic age (Hunt and Slater 2016; Henderson et al. 2023; Nanglu and Cullen 2023).
5. For transparency and scientific reproducibility, record all the sources used to derive absolute ages of fossil tips in the supplementary material associated with the analysis.

#### Open questions

The influence of stratigraphic information over phylogenetic relationships has been debated (Fisher 2008; King 2021). Fossil taxa might be prone to a phylogenetic position more congruent with their age in tip-dating analysis—older fossils might be placed on earlier-diverging branches on the tree, even when morphological characters alone might favor another position (King 2021). Conversely, very young fossil tips might be displaced onto recently diverging branches (Mongiardino Koch et al. 2021). This complex interplay between age of fossil specimens and their morphological characters, as well as their combined effect on topological estimate in the context of tip-dating analyses, needs to be more thoroughly evaluated.

#### General recommendations

We advocate for using temporal age information from the fossil record in tip-dating analyses specifically by

1. explicitly identifying “taxon stratigraphic age range” (“first and last” appearance dates) and “specimen stratigraphic age uncertainty” for age implementation in phylogenetic analyses;
2. utilizing fossil age uncertainty instead of a midpoint or an arbitrary selection of an age interval, for both specimen and species age ranges (Barido-Sottani et al. 2019, 2020); and
3. critically evaluating stratigraphic congruence of the resulting tree upon expertise of the investigated group of organisms.

### Node Calibrations

The age of the earliest assigned fossil of a determined taxon can be used as calibration for selected nodes by specifying minimum and maximum node ages in molecular and tip-dating studies (Kishino et al. 2001; Benton et al. 2009; Parham et al. 2012) (i.e., primary calibration). The determination of the youngest possible age of a taxon selected for calibration comprises the minimum age for the node (i.e., hard minimum), which in turn implicitly also accommodates the error associated with the geochronologic age (i.e., stratigraphic uncertainty of the age of a fossil specimen) (Parham et al. 2012). Conversely, determining the maximum age for fossil calibrations can be contentious. By definition, the soft maximum constraint consists of an age older than the oldest possible record of a lineage in which no fossils are known, but the conditions (ecologic, biogeographic, geologic, and taphonomic) for the existence of such a lineage are met (Parham et al. 2012). Soft maximum boundaries, as well as the shape of the probability distribution associated with the node age, have been argued to be rather subjective (Benton and Donoghue 2007; Parham et al. 2012; Lee and Palci 2015). However, a few model-based approaches can use other fossil occurrences besides the oldest record of a clade to generate calibration densities with soft maxima more objectively (Matschiner et al. 2017; Claramunt 2022; see Marshall 2019 for an in-depth discussion on maximum clade ages).

The main challenge with node calibrations is the clear assignment of the fossil to a crown group, excluding the stem lineage, for which this calibration is applied to. This in turn favors the use of tip-dating methods for time-calibrating topologies due to the approach of simultaneously analyzing molecular, morphological, and stratigraphic data, therefore avoiding the arbitrary selection of age and topological constraints (Lee and Palci 2015). An approach combining node and tip calibrations has been advocated for (O’Reilly and Donoghue 2016), and is indeed not uncommon in empirical tip-dating studies (around one-quarter of surveyed studies also used node calibrations; Fig. 2k). The hard minima of node calibrations contribute to constraining the uncertainty of tip-dating estimates, whereas the latter can contribute to objectively define the maxima of node constraints (see O’Reilly and Donoghue 2016 for details).

In molecular and tip-dating analyses, specific nodes can be alternatively time-constrained by secondary calibrations, which includes the use of results from published molecular dating studies (Shaul and Graur 2002) or biogeographic calibrations (Ho et al. 2015). The former has been advocated as it could improve divergence age estimates if based on primarily robust molecular dating (Hedges and Kumar 2004). However, this approach has been criticized due to an observed shift to younger age estimates, false impression of precision, and lack of methodological approaches to address additional uncertainty in age estimates resulted from the use of secondary calibrations (Shaul and Graur 2002; Graur and Martin 2004; Schenk 2016). The biogeographic calibration approach assumes that geological and climatic events have impacted species and/or populations in a measurable way, and that the age of the biogeographic event can be dated independently (Ho et al. 2015). Biogeographic data constraints may include vicariance, geodispersal, or biodispersal events. This approach is particularly advantageous when alternative calibrations are not known or available (Ho et al. 2015). Similar to the discussion on implementing fossil age for divergence dating analyses, age uncertainty (prior distribution) of the geographic event should be included instead of a point value (Ho et al. 2015).

To avoid potential circularities of node dating, (paleo-)biogeographic information can be integrated in a time-calibrated phylogenetic analysis through process-based biogeographic dating, where observed geographic distributions are the result of range evolution along the phylogeny under a specified model (Landis 2017, 2021; Landis et al. 2018). In some sense, process-based biogeographic dating is to biogeographic data what fossil tip-dating is to paleontological data: a way to directly integrate these data into the phylogenetic analysis, rather than use them indirectly to inform node calibration priors (Landis 2021). Thanks to the modularity of Bayesian phylogenetic analyses, process-based biogeographic dating can in principle be combined with fossil tip-dating, jointly using paleontological and biogeographic data to infer divergence times and topology. To our knowledge, this has never been done yet, and more research in this direction is needed to understand the impact and potential limitations of integrating process-based biogeography into tip-dating.

#### Open questions

The major conundrum with node calibrations using fossil data is the assumption of phylogenetic placement of the fossil to a crown group without uncertainty, an issue that tip-dating tries to avoid (Barido-Sottani et al. 2023). Wrong placement of fossil calibrations can severely impact estimation accuracy, and when in doubt more conservative calibrations are preferable (Barido-Sottani et al. 2023). Combining node and tip calibration can be debatable given the distinct and—to a certain degree—conflicting nature of the two methods. Although the combination of both approaches has been reported to yield more precise divergence time estimates (O’Reilly and Donoghue 2016) and it is commonly used in empirical analyses (Fig. 2k), it is unknown if and how implementation of node calibrations in tip-dating violates the modeling assumptions of the latter.

Objective ways of setting maximum age constraints of node calibrations remain uncertain (Parham et al. 2012; O’Reilly and Donoghue 2016; but see Matschiner et al. 2017; Claramunt 2022), and the sole use of age constraints for time calibration based on primary estimates of molecular studies is dubious (Schenk 2016). Similarly, the effectiveness and reliability of secondary calibrations based on biogeographic events is unclear due lack of a broader implementation (Ho et al. 2015; Landis 2017).

#### General recommendations

If node age constraints are to be applied in the phylogenetic analysis, we recommend the following practices:

1. The specimen-based protocol of Parham et al. (2012) is followed in order to guarantee well-justified fossil calibrations.
2. It is essential to evaluate the phylogenetic relationships of the selected fossil taxa, particularly considering stem versus crown group placement of the fossil, as phylogenetically misplaced calibrations critically compromise divergence time estimates.
3. We recommend the clear identification of stratigraphic age uncertainty for the specimen selected to represent the youngest possible occurrence of a fossil taxon (hard minimum, “last appearance date”).
4. The implementation of biogeographic calibrations should be carefully evaluated for the clade of interest and used in combination with primary calibrations when possible.

### Lineage Evolution Models (Tree Priors)

The very first Bayesian phylogenetic tip-dating analyses either used inconsistent models of lineage evolution, for example, the pure-birth or birth–death process (Pyron 2011), or used non-mechanistic prior distributions such as the uniform prior on node ages (Ronquist et al. 2012). Similarly, these early studies used single point estimates without uncertainty for dates of fossil tips to obtain a temporal perspective. In subsequent years, a considerable effort has been devoted into developing, testing, and exploring more biologically realistic tree prior distributions. Most influential has been the development of the fossilized birth–death (FBD) process (Stadler 2010; Heath et al. 2014), which has been the foundation for continuous model development (Mulvey, Nikolic, et al. 2025). We discuss the model assumptions underlying these tree prior distributions.

### The Fossilized Birth–Death Process

Prior distributions on trees, or tree priors, specify a probability density on the tree topology and divergence times (i.e., node ages). In macroevolutionary tip-dating studies, tree priors must also specify a probability density on sampling times (contrary to tip-dating studies in epidemiology using the coalescent process). Here, we focus exclusively on mechanistic tree priors, namely the FBD process, compared with *ad hoc* tree priors (e.g., a uniform prior on node ages).

**Figure 8.**
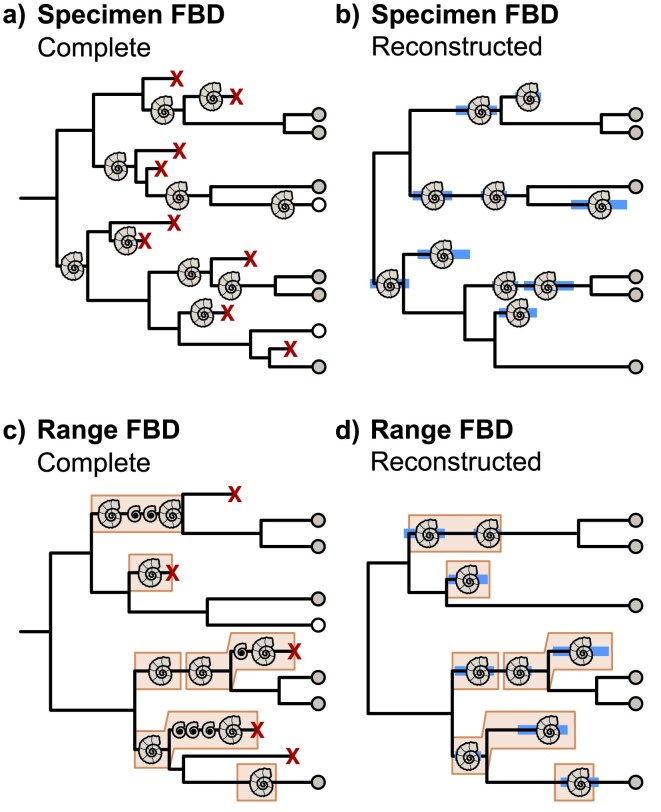
Fossilized Birth–Death (FBD) tree priors. Ammonites symbolize sampled fossils. Terminal dots symbolize extant species (sampled if grayed out, unsampled if white). Xs indicate extinctions. (a) Complete and (b) reconstructed phylogenetic trees under the FBD (or specimen-based FBD, or Sampled Ancestor Birth–Death Serial Sampling) process (Gavryushkina et al. 2014). Bars in the reconstructed tree represent posterior uncertainty in fossil specimen age. (c) Complete and (d) reconstructed phylogenetic trees under the range FBD process (Stadler et al. 2018). Envelopes delimit sampled extinct species. Larger ammonites indicate first and last sampled fossil occurrences of an extinct species. Smaller ammonites represent other sampled fossil occurrences of an extinct species.

The FBD process (Fig. 8) is an extension to the widely used birth–death process (Nee et al. 1994) with serial samples (Stadler 2010; Heath et al. 2014). Specifically, the FBD process assumes exponentially distributed waiting times of speciation, extinction, and sampling events (Fig. 8a), each governed with their own rate parameter (speciation rate, extinction rate, and sampling rate, respectively). An ancestral lineage splits into two descendant lineages at a speciation event and goes extinct at an extinction event. At a sampling event, a specimen of the lineage is sampled and included in the study. Thus, the sampling rate (or fossilization rate) corresponds to a combination of the specimen being preserved as a fossil, the fossil being recovered and being included in the study. In inferences, only the reconstructed tree (Fig. 8b) can be estimated, that is, lineages without samples, either extinct or recent, are removed.

Under the FBD process, a fossil specimen can be sampled either on a lineage leading to other sampled fossil specimens or to sampled extant taxa (in which cases we call it a sampled ancestor; Heath et al. 2014; Gavryushkina et al. 2017), or on a lineage that goes extinct before being sampled again (Fig. 8a). Sampled ancestors are effectively tips with zero-length terminal branches. Tip-dating phylogenetic software can allow to constrain the analysis so that fossils are never recovered as sampled ancestors. Although this is not a rare practice in empirical analyses (Fig. 2i and Supplementary Table S1; Simões, Caldwell, et al. 2020), it is questionable from a modeling perspective as it violates the assumptions behind the process (Gavryushkina et al. 2017).

In addition to speciation, extinction, and fossil sampling rates, another model parameter of the FBD process is the time of origin of the process. Alternatively, the model can be reparameterized with the time of the most recent common ancestor (or root age) (Stadler 2010; Heath et al. 2014). The practical difference between these two quantities is subtle: the time of origin is the time at which a single ancestral lineage originates (i.e., the start of the FBD process); the root age is the time at which the ancestral lineage first splits into two distinct lineages whose descendants have been sampled (i.e., the time of the first reconstructed speciation event). Prior distributions on these parameters can be either uninformative (uniform prior between zero and infinity) or informed by the fossil record similarly to node calibrations (see the “Node Calibrations” subsection and Supplementary Fig. S9). In practical terms, the origin parameter is preferred when fossils, especially sampled ancestors, could be diverging on the stem branch leading to the crown group (see Gavryushkina et al. 2017). If such a scenario is impossible, then the root age parameter is preferred as there is only very little information in estimating the origin age.

The exponential waiting times between speciation events originate from the assumption that each lineage has the same probability to speciate within a tiny time interval $\Delta t$. The same reasoning applies to extinction and fossil sampling events. This assumption of constant speciation, extinction, and sampling probabilities is clearly unrealistic for macroevolutionary studies where most study groups have gone through phases of diversity increase and decrease. Therefore, the constant FBD process has been extended to the *Skyline* FBD process (Gavryushkina et al. 2014; Zhang et al. 2016; Magee and Höhna 2021) with piecewise-constant rates per interval. The assumption of constant rates per interval originated from mathematical convenience as only for this model an analytical solution for the probability density function is known.

#### Modeling speciation, extinction, and sampling rates

Given the Skyline FBD process, some recent work has focused on hyperprior distributions on the speciation, extinction, and sampling rates per interval (e.g., Zhang, Ronquist, et al. 2023). In principle, any number of intervals and prior distribution of rates can be applied. Higher numbers of intervals have the advantage to be more flexible and to approximate more continuous rate functions but come at additional computational costs and possible model overfitting. Recent work has advocated for autocorrelated prior distributions, for example, where the prior distribution of a rate in a given interval depends on the rate in the previous interval (Magee et al. 2020; Zhang, Ronquist, et al. 2023). Examples of such autocorrelated prior distributions are the Gaussian Markov Random Field (i.e., discrete Brownian motion), the Horseshoe Markov Random Field (i.e., discrete Brownian motion with jumps), and the discrete OU process (Zhang, Ronquist, et al. 2023). Such autocorrelated priors allow for flexibility of rates over time while being regularizing, that is, collapse to constant rates in the absence of data, and are recommended to be used with 20–100 intervals (Magee et al. 2020). Thanks to this flexibility of diversification and fossil-sampling rates, the Skyline FBD process can partially compensate for empirical deviations from model assumptions in the sampling strategies for extant and extinct taxa (Zhang, Ronquist, et al. 2023).

#### Incomplete taxon sampling of extant taxa

Most phylogenetic studies, including tip-dating analyses, do not include all extant species of the study group (so-called incomplete taxon sampling). Incomplete taxon sampling can bias diversification rates estimates (Cusimano and Renner 2010; Höhna et al. 2011; Höhna 2014) and divergence times estimates (Ronquist et al. 2016; Zhang et al. 2016). There are several important aspects about incomplete taxon sampling for tip-dating studies to consider. First, in this context, incomplete taxon sampling only refers to extant taxa and not to extinct taxa. Second, the fraction of sampled taxa over the total number of taxa needs to be known for realistic diversification rate estimates. An underestimate of the current diversity leads to either drop in recent speciation rates or spike in recent extinction rates (or the reverse if the present diversity if overestimated). Third, the distribution of sampled taxa needs to be modeled closely, for example, if extant taxa are randomly included or to maximize diversity (Matschiner 2019; Luo et al. 2023). Assuming random sampling in cases where extant taxa have been picked to maximize diversity leads to an overestimation of divergence times, an effect that has been termed “deep root attraction” (Ronquist et al. 2016; Zhang et al. 2016; Matschiner 2019; Luo et al. 2023).

#### Modeling fossil samples

The original implementation of the FBD process (Fig. 8) treats fossil samples as specimens of a lineage (Stadler 2010; Gavryushkina et al. 2014; Heath et al. 2014). Uncertainty in the fossil sample age can be integrated and is usually done by assigning a uniform distribution to the fossil tip obtained from the stratigraphic age range (Drummond and Stadler 2016; Barido-Sottani et al. 2019, 2020). Assuming a constant and equal among lineages fossilization rate, the FBD process requests to include all fossil specimens even if multiple fossil specimen are available for the same extinct species. However, including more than one specimen per extinct species is rarely practiced.

A recent theoretical development of FBD process models has led to the fossilized birth–death-range (FBD-range) process (Figs. 8c and d; Stadler et al. 2018). The FBD-range process additionally models anagenetic speciation—where one species gives rise to another one on the same lineage—and different modes of cladogenetic speciation (budding or asymmetric, and bifurcating or symmetric; Stadler et al. 2018). Thus, the FBD-range process can model the origination and termination of a species along a lineage and therefore use the oldest and youngest occurrence of an extinct species (Fig. 8c). Additionally to currently lacking stable implementations, the FBD-range process introduces the additional challenge that fossil specimens need to be clearly assigned to fossil species (see discussion on morphospecies above).

#### Topological constraints

Finally, the application of topological constraints (i.e., specifying a set of taxa to be monophyletic) has been discussed in the context of FBD analyses (Barido-Sottani et al. 2023). Originally, clade constraints have been commonly applied in fossil phylogenies for instance to fix the phylogenetic position of living species according to the molecular topology, commonly referred as molecular backbone constraint or molecular scaffold (Beck and Lee 2014; Crawford et al. 2015; Lee and Palci 2015; O’Reilly et al. 2015; Darlim et al. 2022). Considering large amount of molecular data in modern data sets, topologies of extant taxa are most likely governed by DNA (Lee and Palci 2015). Therefore, enforcing relationships of extant taxa through a molecular scaffold is an effective alternative that generates topologies compatible to results of analyses combining morphological and molecular data (Lee and Palci 2015; Darlim et al. 2022).

Regarding fossil placement, topological constraints are an efficient approach to incorporate fossils in the topology, especially regarding fragmentary specimens (O’Reilly and Donoghue 2016; Brown and Thomson 2018; Mongiardino Koch et al. 2023). Enforcing constraints for specifying phylogenetic affinities of fossil taxa comes with little computational costs in FBD analyses, as similarly observed with molecular scaffolds. Despite violating the principle of co-estimating divergence times and topology of tip-dating analyses (Lee and Palci 2015; Šmíd and Tolley 2019; Thomas et al. 2020; Barido-Sottani et al. 2023), topological constraints might produce more accurate trees under an FBD analysis (Ronquist et al. 2012; Barido-Sottani et al. 2023; Mongiardino Koch et al. 2023). However, in the context of specifying fossil placement in the topology, this is only possible if well-grounded taxonomic identification and affinities of the fossil taxa are considered, which might not be accessible in many cases in empirical data sets (Barido-Sottani et al. 2023). Incorrect assignment of fossils in node constraints can affect accuracy on results of the phylogenetic analysis, potentially including divergence age estimates (Donoghue and Yang 2016; Brown and Smith 2018; Barido-Sottani et al. 2023; but see Mongiardino Koch et al. 2023). Accordingly, reliability of topological constraints needs to be explicitly justified based on a set of unequivocal synapomorphies and an overview of phylogenetic analyses in a similar best-practices approach that is used for justifying node calibrations (i.e., Parham et al. 2012), and the use of more inclusive node constraints is preferred (Barido-Sottani et al. 2023).

#### Open questions

The FBD process has clearly been a fundamental advance for tip-dating studies. The success of the FBD primarily stems from avoiding to choose *ad hoc* calibration densities and instead coherently models fossil samples in the same process as the tree prior (Heath et al. 2014). However, different assumptions and variants of the FBD process have a striking impact on divergence time estimates (e.g., Ronquist et al. 2016; Zhang, Ronquist, et al. 2023). Importantly, this implies that the choice of prior (i.e., the specific tree prior) has a strong influence on the posterior estimates (the divergence times) and overwhelms the signal in the data. Given that several assumptions of the FBD might be violated, we emphasize to be cautious instead of being overconfident in the result. For example, the FBD process should not be applied to data sets including outgroup taxa as the ingroup and outgroup can have strikingly different sampling fractions. Furthermore, current implementations of the FBD process assume homogeneous speciation, extinction, and fossilization rates among lineages (albeit varying over time, but see Barido-Sottani and Morlon 2025), which is clearly violated for many study systems (e.g., Černỳ et al. 2022). Current tip-dating analyses often cover at least one mass extinction event, which is not modeled in the trajectory of extinction rates (but see Magee and Höhna 2021). Finally, fossil sampling biases, such as differential preservation, Lagerstätten effects, and human interest (Benson et al. 2010), are rarely constant over time and even among lineages. More work on studying the impact of these model violations is needed.

#### General recommendations

We suggest the following for modeling lineage evolution:

1. Specimen versus range FBD: The first question to consider when choosing an appropriate tree prior is to consider whether fossil OTUs represent specimens or extinct species ranges, and thus to choose either the specimen-based FBD or FBD-range process (Fig. 8). Given current practical challenges in the implementation of the FBD-range process, we presently advise to collect fossil data in a way that is compatible with the assumptions of the specimen-based FBD process.
2. Fossil inclusion: Inclusion of as many fossil specimens as possible is desirable (Luo et al. 2020; O’Reilly and Donoghue 2020). If a selection has to be made, a random subsampling is advisable over the preference of simply choosing older fossils to not overestimate divergence times (Matschiner 2019; but see Zhang, Drummond, et al. 2023 for possible benefits of picking older fossils in FBD analyses with continuous characters).
3. Fossil age uncertainty: We strongly recommend to include age uncertainty of fossil samples (Barido-Sottani et al. 2019) and to select the appropriate extant taxon sampling approach.
4. Diversification rate modeling: Given the state-of-the-art, we recommend to use the Skyline FBD process together with regularizing prior distributions on the speciation, extinction, and fossilization rates such as the GMRF, HSRMF, or OU (Magee et al. 2020; Zhang, Ronquist, et al. 2023) with 20–100 time intervals. Even though the Skyline FBD model has shown to produce comparable divergence time estimates compared with the constant-rate FBD, we advocate for the more complex model as it collapses into the simpler model.

## General Conclusions and Outlook

Just a decade after their first introduction, tip-dating approaches have evolved considerably and now represent the state-of-the-art to infer evolutionary relationships and divergence times between extant and extinct organisms (Mulvey, Nikolic, et al. 2025). The modularity of tip-dating analyses provides multilayered possibilities to incorporate different types of data, and design model settings that are appropriate for the data and system analyzed. The flexibility to include extinct and extant organisms, genotypic and phenotypic data, as well as age information, allows for a more integrated reconstruction of evolutionary history. For these reasons, tip-dating is an extremely compelling approach, even though the interaction between its different components can be computationally challenging and conceptually complicated.

**Figure 9.**
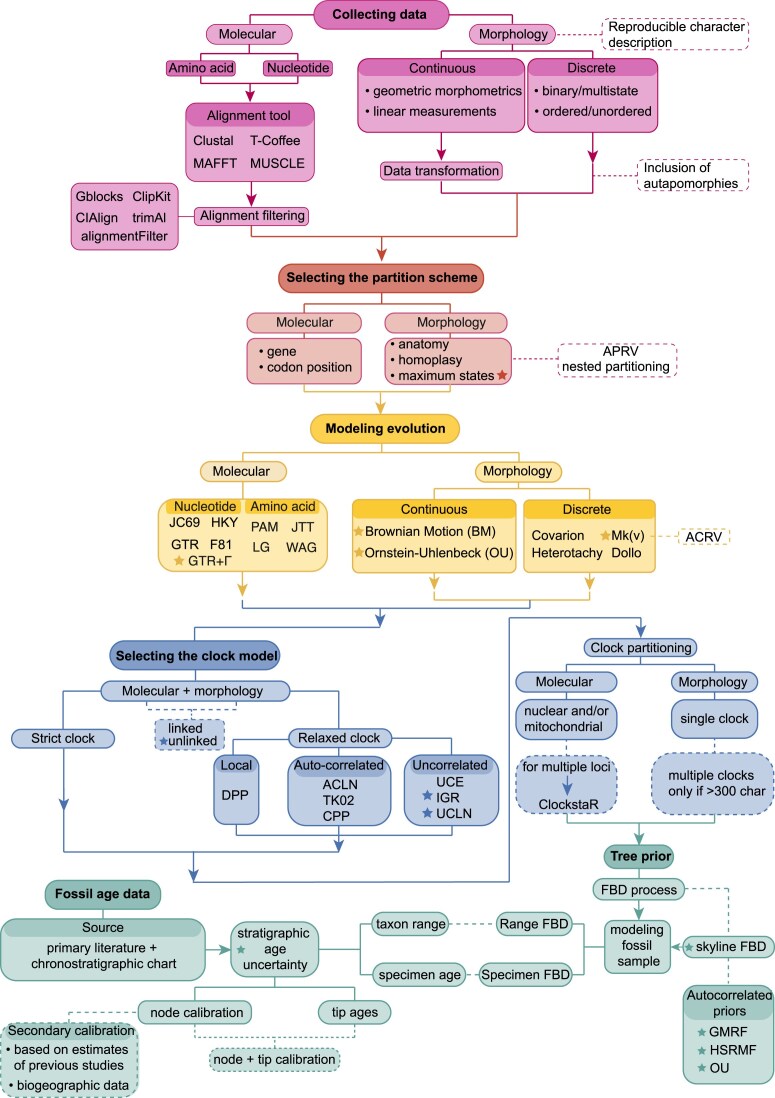
Flowchart representing the steps for setting up a tip-dating analysis. Boxes indicate different data types (morphology and molecular) and alternatives for data treatment, including data partitioning schemes, models of evolution, alternatives for clock models and tree priors, and collection of age information from the fossil record. Star indicates our recommendations (best practices) based on what have been discussed along the manuscript. Dashed boxes indicate additional recommendations for data treatment.

In this practical guide, we covered the three main components of a Bayesian phylogenetic tip-dating study: models of molecular evolution, models of morphological evolution (for both discrete and continuous data), and models of lineage evolution. We presented the state-of-the-art together with some open questions and general recommendations (summarized in Fig. 9). We generally recommend choosing more complex, parameter-rich models over simplistic ones—for example, relaxed over strict clocks, ACRV over equal-rate character models, and partitioned over unpartitioned approaches. The most common reason to choose simpler over more complex models is time-related, as a tip-dating analysis using complex models might not be feasible. Nevertheless, we encourage to try complex models as much as computationally possible. In principle, model selection procedures should be able to select the most appropriate models for a given data set. Unfortunately, tip-dating studies face several new challenges regarding model selection, including (i) a nonstandard separation between fossils as belonging to the data (likelihood) or parameters (prior) (May and Rothfels 2023), (ii) inapplicability due to changes in the assumption of the underlying data when changing the state space (Khakurel et al. 2024; Mulvey, May, et al. 2025), and (iii) significantly increased computational burden to already time-consuming analyses. It might therefore be advisable to perform several analyses under different model/prior assumptions. Finally, we stress out the importance of always checking whether posterior parameter estimates are just recapitulating effective prior distributions to evaluate if input data are informative enough for the chosen models.

There exist several overarching recommendations to improve tip-dating analyses. First, data collection should always be comprehensible and well documented. Particularly in the case of morphological characters, reproducible and explicit character descriptions and state definitions are essential. Moreover, it is crucial that input data for a tip-dating analysis are collected while keeping in mind the several assumptions behind chosen models—for example, sampling autapomorphies if applying the Mkv model for morphological characters, or sampling fossil specimens (rather than species) for the specimen-based FBD model. Second, transparent ways of decision-making for software and modeling choices and reasoning behind all parameter settings ensure reproducibility for future studies. Thus, we highly recommend to publicly upload all data and script files used at every step of the analysis, as well as documenting in detail each of those steps. Additionally, researchers should minimize as much as possible manual manipulation of software output (e.g., alignments).

As highlighted throughout this contribution, although some components of tip-dating analyses are already well-established (e.g., molecular substitution models), others are still in their infancy or have received considerably less attention (e.g., morphological substitution and clock models, morphological partitioning). Efforts in model development in key areas will be fundamental to advance tip-dating approaches. Besides the development of new models, substantial progress will be made by evaluating through posterior predictive analyses whether available models are adequate or not for empirical data—a yet underexplored research avenue (Brown 2014; Brown and Thomson 2018; Mulvey, May, et al. 2025).

Despite the limitations that we pointed out in this paper, tip-dating approaches hold exceptional promise for the future of phylogenetics and systematics, and are likely to become more and more relevant within the broader context of evolutionary biology, paleobiology, and geobiology. We argue that a closer collaboration between researchers involved in phylogenetic software development and researchers working on empirical systems (paleontological and neontological) will be beneficial for both, allowing for the development of more realistic models whose assumptions fit more closely to the nature of empirical data, and conversely empowering empirical researchers to better design their research. We hope that our contribution represents a decisive step in this direction.

Ultimately, we point out that no matter how sophisticated the models employed in tip-dating analyses will become, the crucial factor of these analyses remains the availability of high-quality, densely sampled data. Because molecular data collection has already undergone a revolution that has drastically improved their quantity, quality, and cost-efficiency, significant progress in tip-dating studies will hang on efforts to increase and advance morphological data collection and global sampling of paleontological data. Under this lens, for tip-dating the sky is the limit, yet the truth lies in rocks down below.

## Supplementary Material

syaf050_Supplemental_File
