# Supplementary material for: Practical Guide and Review of Fossil Tip-Dating in Phylogenetics
Source: Syst Biol. 2025 Sep 24;75(1):156–92. doi: 10.1093/sysbio/syaf050 (PMC12805671; doi:10.1093/sysbio/syaf050)
Supplement: syaf050_Supplemental_File [file syaf050_supplemental_file.pdf]

Supplementary Document to ‘Practical Guide and Review of Tip-Dating in Phylogenetics’

Nicola S. Heckeberg, Alessio Capobianco, Basanta Khakurel, Gustavo Darlim, and Sebastian Höhna

This document is a supplement to the manuscript ‘Practical Guide and Review of Tip-Dating in Phylogenetics’. It consists of a table with brief description about some common molecular substitution model and several figures obtained from a literature survey on tip-dating studies from 2011 to 2023.

Supplementary Table S2

Additional figures summarizing the publication survey

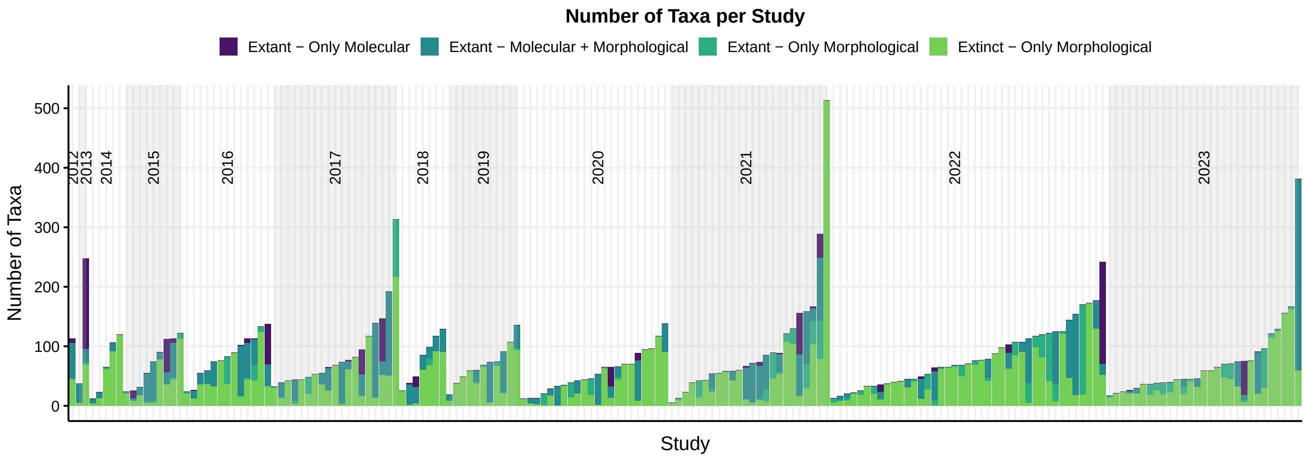

Figure S1: Number of taxa per study. The bars represent the total number of taxa in the study and the corresponding colors indicate the proportion of taxa with molecular sequence and/or morphological characters. Each alternating shaded region represents the corresponding year that the studies are published.

Table S2: A table with some of the most commonly used molecular substitution models and a brief description about the models. The data type indicates nucleotide (NT) or Amino Acid (AA) datasets.

Substitution Model	Data Type	Description	References
JC69 (JC)	NT	Rate of nucleotide substitution is same for all pairs of nucleotides.	(Jukes and Cantor, 1969)
K80 (K2P)	NT	Transition and transversion rates allowed to vary but the nucleotide frequency is assumed to be equal.	(Kimura, 1980)
F81	NT	Exchangeabilties are assumed to be equal and the base frequencies are unequal.	(Felsenstein, 1981)
K81 (K3P)	NT	Different transversion rates, same transition rate, and equal base frequencies.	(Kimura, 1981)
HKY (HKY85)	NT	Transition and transversion rates are different and the base frequencies are unequal.	(Hasegawa et al., 1985)
TN93	NT	Same transversions rates, different transition rates and the base frequencies are unequal.	(Tamura and Nei, 1993)
SYM	NT	Different rates between the nucleotides and equal base frequencies.	(Zharkikh, 1994)
GTR	NT	Different rates between the nucleotides and base frequencies are unequal.	(Tavaré, 1986)
PAM	AA	Based on observed amino acid mutations in closely related proteins and extrapolates probabilities of change over longer evolutionary times.	(Dayhoff et al., 1978)
JTT	AA	Empirical substitution matrix derived from a larger and more diverse dataset of protein alignments than PAM matrices.	(Jones et al., 1992)
WAG	AA	Matrix derived using maximum likelihood method that considers the phylogenetic relationships within protein families to estimate amino acid replacement rates for globular proteins.	(Whelan and Goldman, 2001)
LG	AA	Matrix estimated using maximum likelihood approach from large database of alignments, incorporating site heterogeneity in evolutionary rates.	(?)

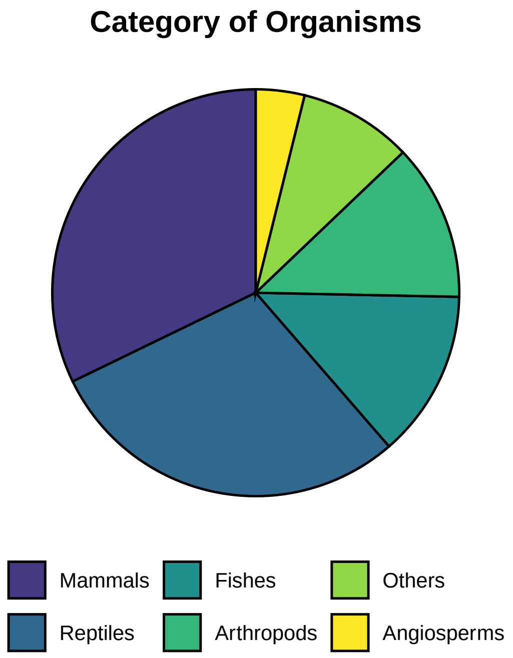

Figure S2: Group of organisms most commonly studied in tip-dating studies.

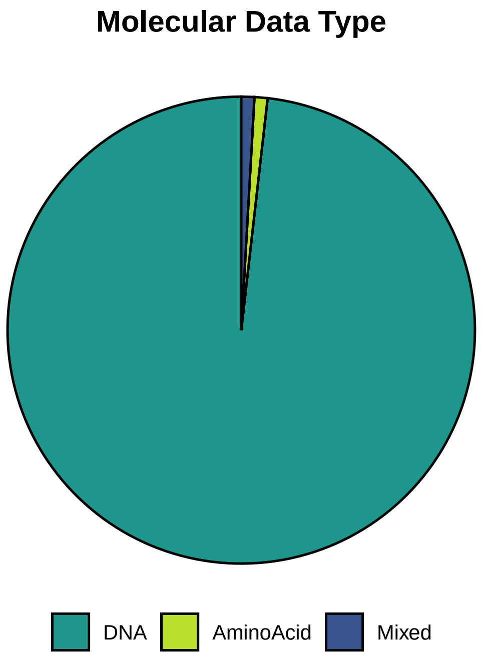

Figure S3: Type of molecular data used in tip-dating studies. 'Mixed' indicates the use of both Amino acid and DNA sequences.

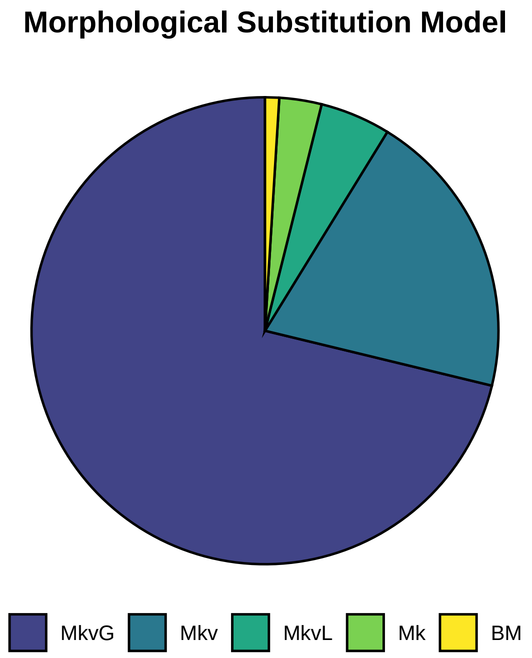

Figure S4: A Pie chart showing the most commonly used morphological substitution model. MkvG (Mk model with ascertainment bias correction and ACRV with Gamma distribution), Mkv (Mk model with ascertainment bias for variable sites only), MkvL (Mk model with ascertainment bias correction and ACRV with Lognormal distribution), Mk (Markov k model), and BM (Brownian Motion).

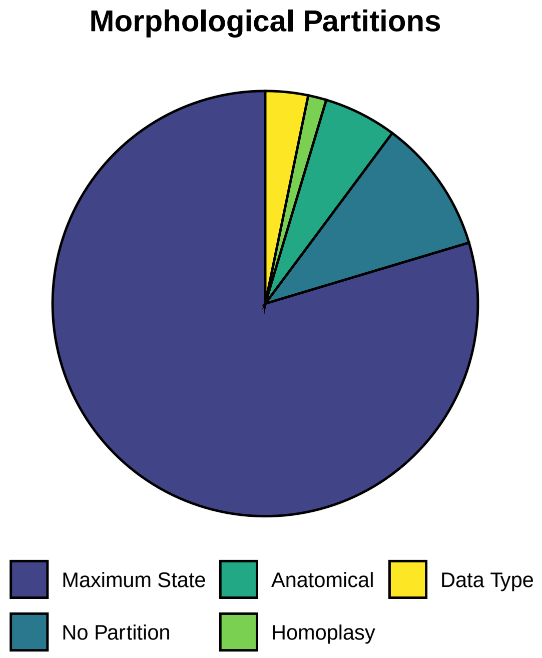

Figure S5: Partitioning schemes applied to morphological data in tip-dating studies. See manuscript for the details about the partitioning approaches.

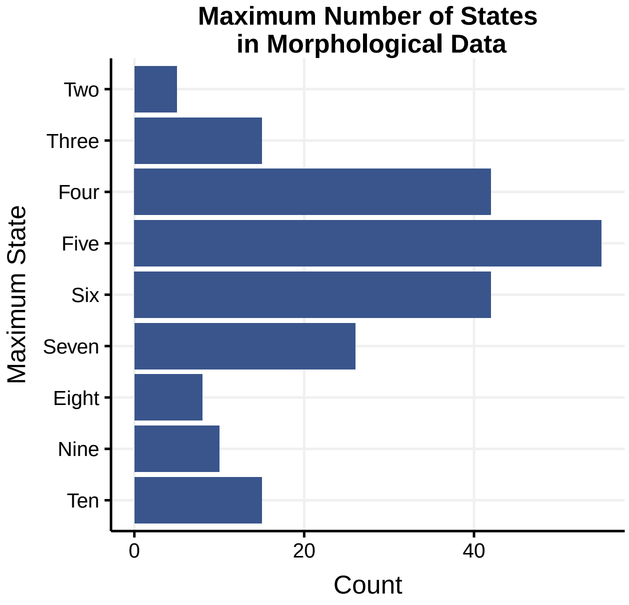

Figure S6: Bar chart showing the maximum number of states used in a morphological matrix.

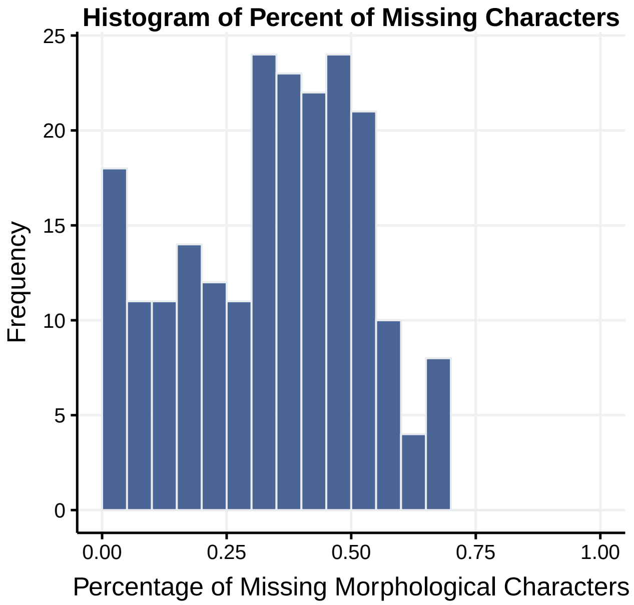

Figure S7: Histogram with the percentage of missing morphological characters in a morphological matrix.

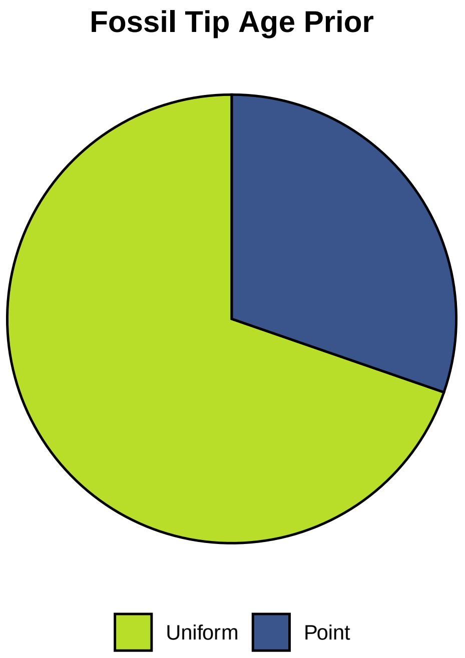

Figure S8: Fossil tip priors used in tip-dating studies.

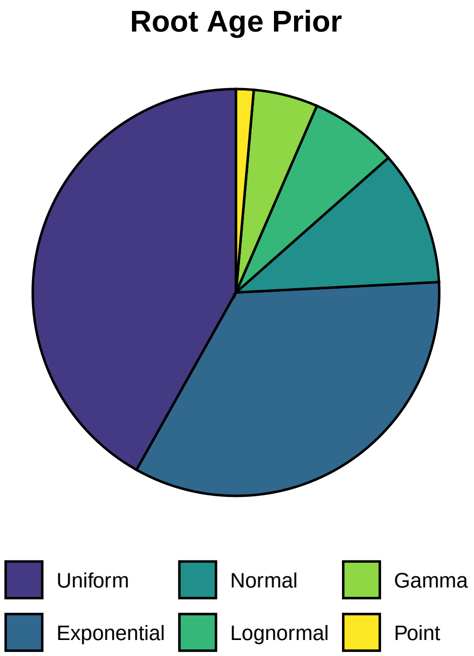

Figure S9: Root age prior in tip-dating studies.
